# Transcriptome Analysis Reveals Common and Differential Response to Low Temperature Exposure Between Tolerant and Sensitive Blue Tilapia (*Oreochromis aureus*)

**DOI:** 10.3389/fgene.2019.00100

**Published:** 2019-02-26

**Authors:** Tali Nitzan, Fotini Kokou, Adi Doron-Faigenboim, Tatiana Slosman, Jakob Biran, Itzhak Mizrahi, Tatyana Zak, Ayana Benet, Avner Cnaani

**Affiliations:** ^1^Institute of Animal Science, Agricultural Research Organization, Rishon LeZion, Israel; ^2^Department of Life Sciences, Ben-Gurion University of the Negev, Beer-Sheva, Israel; ^3^The Aquaculture Research Station, Ministry of Agriculture and Rural Development, Dor, Israel

**Keywords:** carbon metabolism, cold tolerance, Oreochromis, selective breeding, tilapia, transcriptome

## Abstract

Tilapias are very important to the world's aquaculture. As befitting fish of their tropical origin, their distribution, and culture practices are highly affected by low temperatures. In this study, we used genetic and genomic methodologies to reveal pathways involved in the response and tolerance of blue tilapia (*Oreochromis aureus*) to low temperature stress. Cold tolerance was characterized in 66 families of blue tilapia. Fish from cold-tolerant and cold-sensitive families were sampled at 24 and 12°C, and the transcriptional responses to low-temperature exposure were measured in the gills and liver by high-throughput mRNA sequencing. Four hundred and ninety four genes displayed a similar temperature-dependent expression in both tolerant and sensitive fish and in the two tissues, representing the core molecular response to low temperature exposure. KEGG pathway analysis of these genes revealed down-regulation of focal-adhesion and other cell-extracellular matrix (ECM) interactions, and up-regulation of proteasome and various intra-cellular proteolytic activities. Differential responses between cold-tolerant and cold-sensitive fish were found with genes and pathways that were up-regulated in one group and down-regulated in the other. This reverse response was characterized by genes involved in metabolic pathways such as glycolysis/gluconeogenesis in the gills and biosynthesis of amino-acids in the liver, with low temperature down-regulation in tolerant fish and up-regulation in sensitive fish.

## Introduction

Environmental stressors disrupt homeostasis and are harmful to the physiological function of organisms. Environmental temperature is one of the main factors that drove a wide array of evolutionary adaptations, including the division of animals into homeotherms (endotherms), like mammals and birds, and poikilotherms (ectotherms), like reptiles, amphibians, and fish. Fish species inhabit waters of a wide range of temperatures, from below 0°C in the Antarctic ocean to above 40°C in East African lakes (DeVries and Wohlschlag, 1969; Reite et al., 1974). However, each fish species can survive within a limited range of temperatures, and the response to fluctuations in environmental temperature is a crucial factor of its fitness and survival (Schulte et al., 2011). There are various biological components and pathways that respond to changes in the environmental temperature. These include alteration of enzymatic activity and efficiency, membrane permeability, gas solubility, particle diffusion rates, and notably, metabolic rate (Battersby and Moyes, 1998; Itoi et al., 2003).

Information about the genes and biological pathways that affect the ability of fish to acclimate and function in a wide range of environmental and body temperatures is scarce. Several studies, on different fish species, used transcriptomic approaches in order to gain broad view of genes involved in the response and acclimation to low temperatures (Gracey et al., 2004; Long et al., 2012, 2013; Mininni et al., 2014; Hu et al., 2016). These studies pointed to different tissues involved in the physiological response and acclimation to low temperature stress, sometimes with tissue-specific responses. In addition, certain pathways, such as mitochondrial function, lipid and carbohydrate metabolism, anti-oxidant response, apoptosis, RNA processing, and protein catabolism, were found to have temperature-dependent regulation.

Tilapiine fishes of the family Cichlidae originate from the tropical and subtropical parts of Africa, with colonization into the Middle East through the Great Rift Valley (Trewavas, 1983). During the twentieth century, tilapias were introduced to Asia, South and North America and are now highly important in global aquaculture production (FAO data at: www.fao.org/fishery/culturedspecies/Oreochromis_niloticus/en). Reflecting their tropical origin, the optimal temperature for growth of most tilapiine species lies within the range of 20–30°C and reproduction and feeding are usually suppressed at temperatures below 20°C. Variation in the lower lethal temperature has been observed among different tilapiine species (Wohlfarth and Hulata, 1983), with blue tilapia (*Oreochromis aureus*) being one of the most cold-tolerant species (Cnaani et al., 2000). Within species variation in cold tolerance has been attributed to acclimation, physiological stage, environmental factors, and to genetic effects.

Thermal tolerance is a quantitative trait of considerable economic importance in several fish species, including tilapia. Linkage analyses using microsatellites markers resulted in QTL of minor effect (Perry et al., 2001; Cnaani et al., 2003). Several studies attempted to track the inheritance pattern and genetic basis of tilapia's cold tolerance (Cnaani et al., 2000, 2003; Charo-Karisa et al., 2005; Thodesen et al., 2013; Nitzan et al., 2016), however, mechanisms that underlie the within-species variation of cold tolerance remain unknown. Identification of such pathways can open new directions to improve brood-stocks and provide insights into the nature of environmental tolerance and adaptation.

In this work, we used a population with distinct tolerant and sensitive families, obtained through selective breeding, to characterize within-species variation. We then performed transcriptome analysis to compare the gills and liver transcriptome responses to low temperature challenges between cold tolerant and sensitive blue tilapia. We characterized the gene expression pattern which is the general temperature-dependent response common among different fish and tissues, as well as the tolerance-based differences between the cold tolerant and sensitive fish.

## Materials and Methods

### Animals and Experimental Conditions

**Figure 1:**
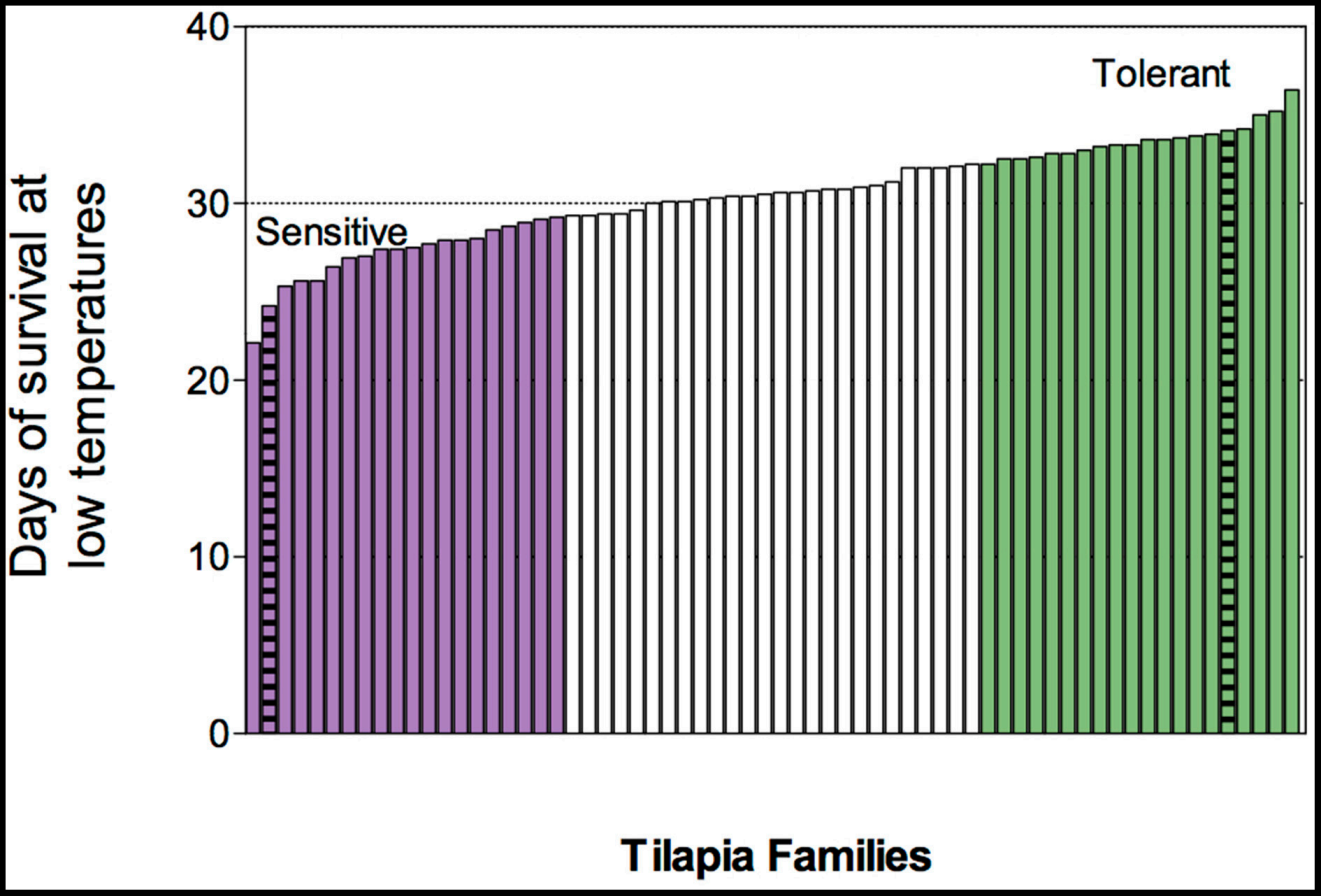
Phenotypic distribution of cold tolerance, as measured in median survival day, of 66 blue tilapia families that were challenged under declined temperature regime (*n* = 10–15 fish/family). Resistant families are highlighted in purple and tolerant families in green. Families used for transcriptome sequencing are marked with stripes.

The fish used in this study were from an Israeli strain of *O. aureus* that is under an ongoing selective breeding program at the Dor Aquaculture Research Station (Zak et al., 2014). Spawns were conducted in pairs-mating to obtain 66 families. Offspring of each family were grown in individual tanks, until being marked with a specific family code using sub-dermally injected dyes when the fish were about 4 months old and weigh ~40 g. In order to determine a cold-tolerance value for each family, 10–15 fish from each family faced a cold-challenge experiment, similar to our previously described study (Nitzan et al., 2016). The 20 families with the highest mean of survival days were considered to have high cold tolerance and the 20 families with the lowest mean of survival days were considered to have low cold tolerance (Figure 1). Sibling of the challenged fish, from three families with high cold tolerance and three families with low cold tolerance, were kept in a common 1,000 L tank at 24°C. After 1 week, half of the fish were transferred to a 600 L tank within a temperature-controlled room (with thermostat within the tank), where the water temperature was reduced from 24°C at a rate of 1°C/day and maintained at 12°C for 2 days. The fish were not fed throughout the challenge, dissolved oxygen levels were kept at above 90% saturation, while ammonia and nitrite levels were not detectable.

This study was approved by the Agricultural Research Organization Committee for Ethics in Using Experimental Animals and was carried out in compliance with the current laws governing biological research in Israel (Approval number: 146/09IL).

### Tissue Collection and RNA Extraction

Seven fish from each family were sampled at 24°C, and seven at 12°C. Gills and liver samples were taken and kept in RNAlater buffer (Qiagen, Hilden, Germany) at −20°C until use. mRNA was extracted from the tissue samples using TRIzol® reagent (Thermo Fisher Scientific, Waltham, MA, United States), and purified to remove DNA contamination using the TURBO DNA-free^TM^ kit (Invitrogen, Carlsbad, CA, United States). RNA concentration and quality were determined using an Epoch Microplate Spectrophotometer (BioTek, Winooski, VT).

### Transcriptome Sequencing

For each temperature, 2 μg RNA from gills and liver samples from three cold tolerant (Family 480) and three cold sensitive (Family 740) fish were sent on dry-ice to the Technion Genome Center (Haifa, Israel). Twenty-four libraries were prepared and sequenced on three lanes on an Illumina Hi-Seq 2500 device.

### Quantitative Real-Time PCR

Specific primers for quantitative real-time PCR (qPCR) were designed for eight genes that were found to have temperature-dependent expression in the Next-Generation Sequencing (NGS) analysis, within a pathway that was differentially expressed between cold-tolerant and cold-sensitive fish: *aldoaa, gpib, pfkma, pgam2, gpia, ldha, tbiB, pgam1a* (the sequences of the primers are listed in Table S1). mRNA from the gills of nine cold tolerant and nine cold sensitive fish (three fish from each family) was reverse transcribed using the Verso cDNA kit (Thermo Fisher Scientific). Amplification reactions were performed using ABsolute Blue SYBR Green ROX mix (Thermo Fisher Scientific), in a 10 μl reaction volume, with primers at 700 nM, on a Rotor-Gene Q real-time PCR (Qiagen). All reactions were performed as follows: 95°C for 15 min, followed by 35 cycles of 95°C for 15 s, 60–58°C for 30 s, and 72°C for 15 s. Relative expression was calculated using ΔΔCt, with the geometric mean of Elongation Factor 1 (EF-1) and β-actin as reference genes. Significance of differential expression between temperatures was analyzed using the Wilcoxon 2-sample non-parametric test.

### Bioinformatic Analyses

The tilapia Illumina sequences were analyzed as previously described (Ronkin et al., 2015), using the TopHat program to map the reads to the Nile tilapia reference transcriptome (ftp://ftp.ncbi.nlm.nih.gov/genomes/Oreochromis_niloticus/), and the Cufflinks and Cuffdiff software tools (Trapnell et al., 2012) for transcript quantification and differential expression analysis between the two temperatures. Transcripts with at least a 2-fold change in response to temperature (at *q* < 0.05, with FDR adjustment of *p*-values) were regarded as significantly up- or down-regulated. Functional annotation of tilapia transcripts with significant temperature-dependent expression was extended using PANTHER (http://www.pantherdb.org/), based on gene ontology (GO) categories assigned to the human or zebrafish orthologs. The database for annotation, visualization and integrated discovery (DAVID) web software (http://david.abcc.ncifcrf.gov/home.jsp) was used for the functional analysis of KEGG biological pathway enrichment (regarded as significantly up- or down-regulated at adjusted *p*-value of 0.05). Adonis implementation of Permanova (vegan R package Anderson, 2001 was used for comparison between groups for clustering analysis using the Jaccard distance matrix (presence/absence of genes).

## Results

### Cold Tolerance Variation

Low temperature exposure of the 66 challenged families that were phenotyped for their mean survival day resulted in individual fish mortality on a range from days 4 to 38, and a normally distributed family mean survival day on a range from 22 to 36 days (Figure 1). Based on this distribution, we further chose three sensitive and three tolerant families for the transcriptome analysis: Families 720, 730, and 740 had an average survival of 25.6, 22.1, and 24.2 days under the temperature reduction regime, respectively, thus considered as cold-sensitive. Families 430, 460, and 480 had an average survival of 33.9, 34.2, and 34.1 days, respectively, thus considered to be cold-tolerant.

### Transcriptome Sequencing

Raw RNA-Seq sequences were deposited in the SRA database (accession numbers SRR7976381 to SRR7976404 under project PRJNA419688). Overall, 496,843,855 reads obtained from 24 libraries. After quality control, 441,882,583 clean reads (89%) remained for further analysis. From these clean reads, 70.6% were mapped to the tilapia reference genome, similar to mapping rate in previous tilapia's transcriptome analyses (Ronkin et al., 2015; Tao et al., 2018). Information concerning the libraries are listed in Table S2.

### The General Transcriptome Response Blue Tilapia to Temperature Decline

**Figure 2:**
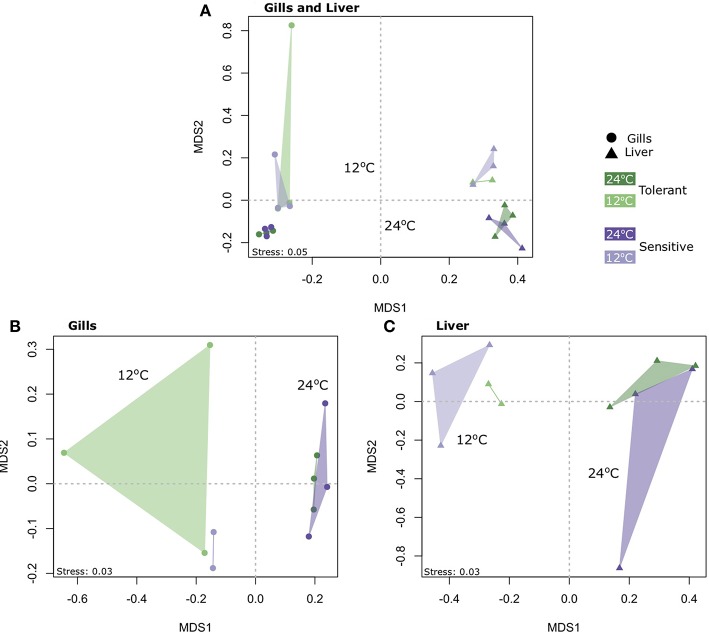
Non-metric multidimensional scaling (NMDS) of gene expression profiles, based on Jaccard similarity (presence/absence of genes), in **(A)** both the gills and liver of cold-tolerant and cold-sensitive fish, **(B)** only the gills, and **(C)** only the liver, at warm (24°C) and cold (12°C) water.

Concerning the number of genes expressed (FDR<0.05 and 2-fold expression difference), Permanova analysis (Table S3A) and clustering using Non-metric multidimensional scaling (NMDS) (Figure 2A) showed that tissue and temperature were the major factors affecting the variance in our dataset. In order to separate the tissue effect, which seems to have the largest affect, we performed Permanova and NMDS analysis for each tissue. Our results showed that temperature was the main factor affecting the gene expression and not the genetic line, with significant clustering according to temperature for both the gills (F_Temperature_ = 3.53, *P* = 0.002) and the liver (F_Temperature_ = 4.39, *P* = 0.005) (Figures 2B,C and Tables S3B, S3C).

**Figure 3:**
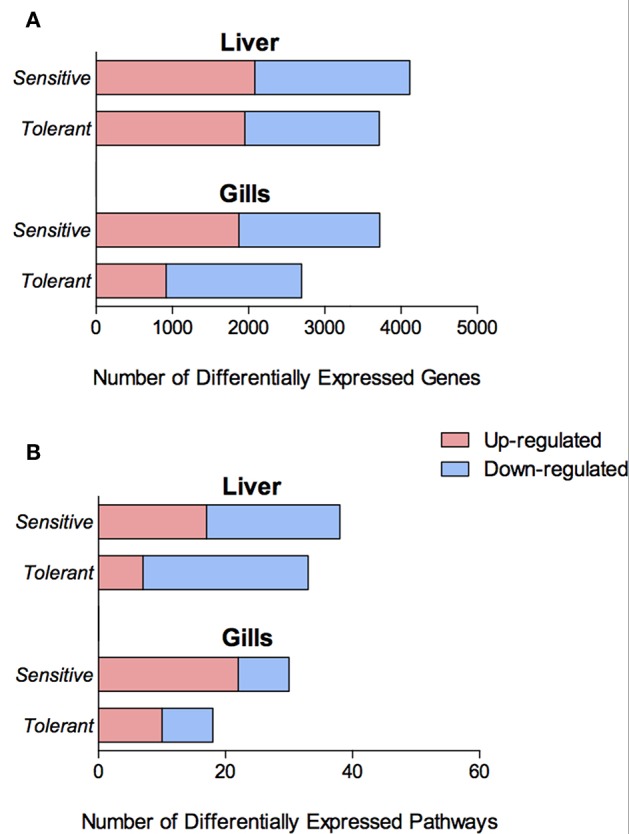
Number of Differentially Expressed Genes (DEGs) **(A)** and differentially enriched KEGG pathways **(B)** in the gills and liver of the cold tolerant and sensitive families, showing both up- (red) and down-regulation (blue).

Exposure to cold temperature led to a total of 2,696 and 3,721 temperature-dependent differentially expressed genes (DEGs) in the gills of families 480 (cold-tolerant) and 740 (cold sensitive), respectively. Additionally, 3,714 and 4,114 temperature-dependent DEGs were found in the liver of families 480 and 740, respectively. In both organs, more genes were up- and down-regulated in the sensitive fish than in the tolerant, 38% more in the gills and 11% more in the liver (Figure 3A).

More specifically, in the gills, 1,590 temperature-dependent DEGs (657 up-regulated and 933 down regulated) were common between the sensitive and tolerant families. In the liver, there were 2,186 temperature-dependent DEGs (1,104 up-regulated and 1,082 down regulated) that were common between the sensitive and tolerant families. Of these, 494 genes (314 up-regulated and 180 down-regulated) were shared between the two tissues (Figure S1). These genes represent the core response of blue tilapia to low temperature exposure (list of these genes in Table S4). KEGG pathway analysis of these genes revealed significant (*P* < 0.05) down-regulation of focal-adhesion and other cell-ECM interactions, as well as up-regulation of proteasome, various intra-cellular protein processing activities, RNA transport and degradation (Table 1).

**Table 1:** Temperature-dependent KEGG pathways observed in more than one family or tissue (↑ for up-regulation and ↓ for down-regulation with *P*-value for each one).

**Pathway**	**Gills**	**Gills**	** Liver**	**Liver**	**Class**	**Function**
**Ribosome biogenesis in eukaryotes**	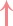 **5e-12**	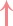 **4e-18**	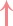 **4e-13**	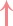 **8e-8**	Genetic information processing	Translation
**RNA transport**	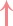 **3e-4**	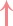 **2e-6**	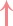 **2e-6**	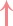 **5e-4**	Genetic information processing	Translation
Ribosome	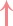 2e-2	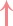 5e-4			Genetic information processing	Translation
Splicosome	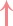 8e-6	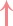 1e-6		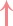 2e-6	Genetic information processing	Transcription
RNA polymerase		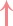 9e-4	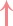 5e-3	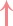 4e-4	Genetic information processing	Transcription
**Proteosome**	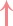 **6e-10**	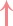 **3e-20**	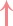 **8e-16**	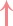 **4e-15**	Genetic information processing	Folding, sorting, and degradation
**Protein processing in endoplasmic reticulum**	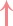 **4e-2**	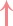 **8e-3**	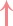 **8e-3**	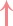 **8e-8**	Genetic information processing	Folding, sorting, and degradation
**RNA degradation**	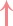 **5e-2**	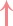 **7e-3**	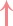 **2e-2**	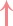 **4e-4**	Genetic information processing	Folding, sorting, and degradation
**ECM receptor interaction**	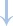 **9e-14**	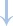 **3e-15**	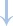 **5e-3**	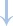 **2e-4**	Environmental information processing	Signaling molecules and interaction
Cytokine-cytokine receptor interaction	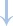 1e-2	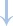 1e-4			Environmental information processing	Signaling molecules and interaction
Jak STAT signaling pathway		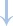 4e-3		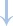 3e-2	Environmental information processing	Signal transduction
**Focal adhesion**	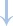 **1e-10**	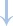 **2e-10**	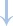 **1e-1**	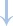 **4e-5**	Cellular processes	Cellular community—eukaryotes
Regulation of actin cytoskeleton	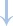 5e-5	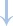 7e-3			Cellular processes	Cell motility
Peroxisome			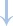 7e-5	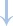 1e-2	Cellular processes	Transport and catabolism
Vascular smooth muscle contraction	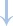 2e-2	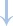 2e-2			Organismal systems	Circulatory system
**Carbon metabolism**	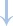 **4e-2**	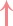 **4e-3**	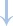 **2e-7**	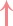 **1e-2**	Metabolism	Overview
**Biosynthesis of amino acids**		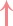 **2e-2**	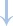 **5e-2**	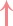 **4e-2**	Metabolism	Overview
Oxidative phosphorylation	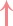 1e-2	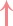 1e-22		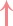 7e-6	metabolism	Energy metabolism
**Pyrimidine metabolism**	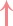 **2e-3**	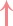 **3e-3**	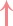 **2e-2**	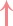 **2e-2**	Metabolism	Nucleotide metabolism
Purine metabolism		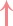 3e-2		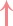 3e-2	Metabolism	Nucleotide metabolism
**Glycolysis/Gluconeogenesis**	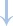 **5e-2**	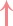 **3e-3**			metabolism	Carbohydrate metabolism
Pentose and glucuronate interconversions			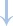 2e-4	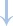 3e-2	Metabolism	Carbohydrate metabolism
Glyoxylate and dicarboxylate metabolism			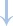 5e-6	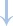 4e-5	Metabolism	Carbohydrate metabolism
Arachidonic acid metabolism			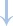 5e-2	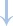 2e-3	Metabolism	Lipid metabolism
Tyrosine metabolism			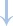 4e-2	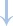 5e-2	Metabolism	Amino acid metabolism
Tryptophan metabolism			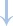 3e-3	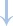 1e-2	Metabolism	Amino acid metabolism
Arginine and proline metabolism			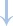 1e-3	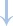 1e-2	Metabolism	Amino acid metabolism
Histidine metabolism			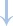 2e-4	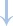 7e-3	Metabolism	Amino acid metabolism
Glycine, serine and threonine metabolism			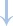 2e-7	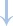 4e-5	Metabolism	Amino acid metabolism
beta-Alanine metabolism			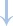 7e-4	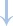 1e-2	metabolism	Metabolism of other amino acids
Glutathione metabolism			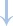 4e-3	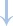 1e-2	Metabolism	Metabolism of other amino acids
Drug metabolism—cytochrome P450			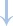 2e-6	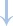 2e-2	Metabolism	Xenobiotics biodegradation and metabolism
Drug metabolism—other enzymes	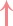 3e-2		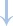 2e-2		Metabolism	Xenobiotics biodegradation and metabolism

*Gills and liver of tolerant fish are highlighted in green and of sensitive fish in purple. Common and differential pathways are in bold letters. Shared pathways between all samples (the core response) are highlighted in red for up-regulation and blue for down-regulation. Pathways with reverse response between resistant and tolerant fish are highlighted in orange*.

### Differential Response Between Cold Tolerant and Sensitive Fish

**Figure 4:**
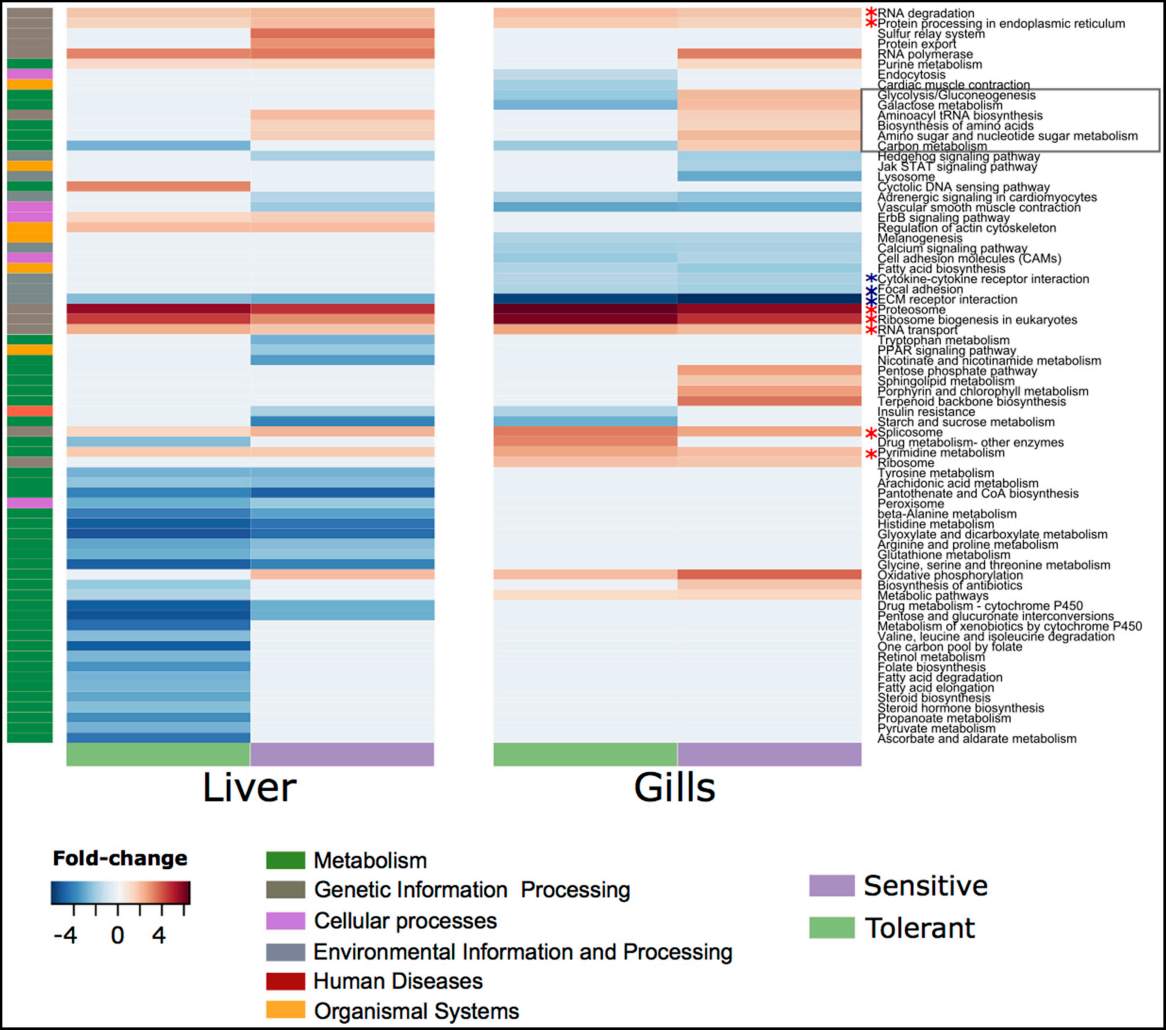
Up- and down-regulated fold changes belonging to different KEGG pathways in the gills and liver of cold tolerant and sensitive fish.

**Figure 5:**
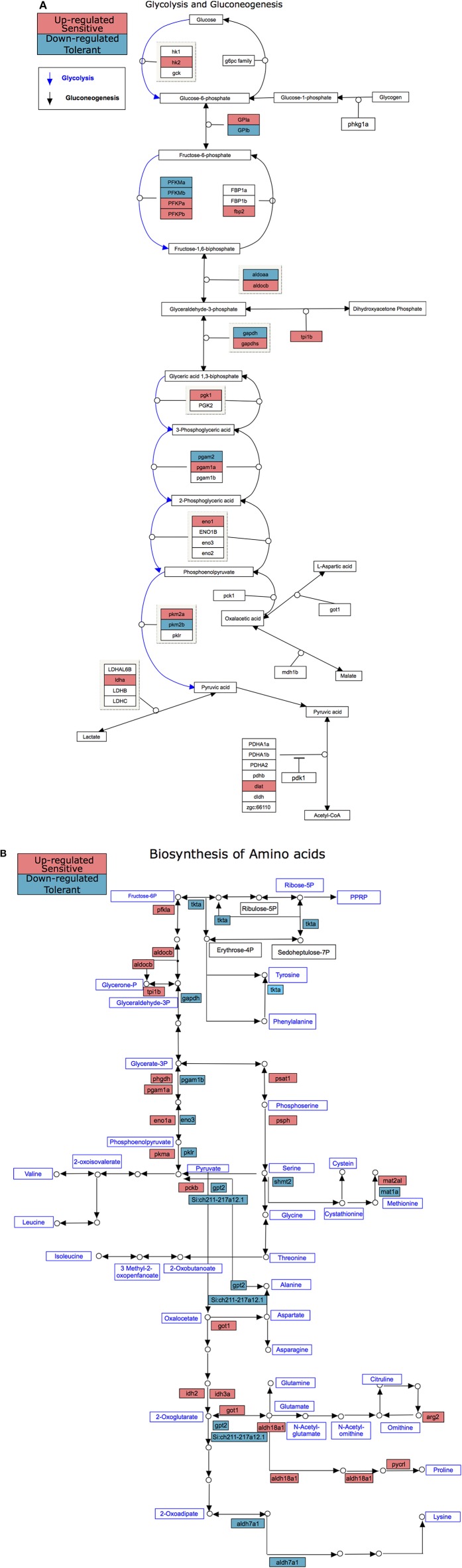
KEGG pathways with differential temperature-dependent regulation between cold sensitive and cold tolerant blue tilapia, glycolysis/gluconeogenesis in the gills **(A)** and biosynthesis of amino-acids in the liver **(B)**. Up-regulated genes in these pathways were found only in the cold sensitive fish and are marked in red, whereas down-regulated genes in these pathways were found only in the cold tolerant fish and are marked in blue.

There were more genes with temperature-dependent expression in the sensitive fish than in the tolerant (Figure 3A). This effect was similar for temperature-dependent enriched KEGG pathways (Figure 3B). Other than the difference in the number of genes and pathways with temperature-dependent differential expression, KEGG analysis revealed an opposite regulation of biological pathways between the cold-sensitive and cold-tolerant fish, with low temperature down-regulation in tolerant fish and up-regulation in sensitive fish (Figure 4, marked in black box). This opposite response was noted for genes involved in metabolic pathways, glycolysis/gluconeogenesis in the gills and biosynthesis of amino-acids in the liver (Figure 5). It is worth noting that no specific gene showed an opposite response between the cold sensitive and cold tolerant fish, rather, different genes within the pathway (sometimes paralogous genes) that were either up- or down-regulated. Comparison of the basal expression (transcripts levels at 24°C) of these genes revealed no differences between the cold-sensitive and cold-tolerant fish (Table S5).

### Validation of Transcriptome Sequencing Results Using qPCR

**Figure 6:**
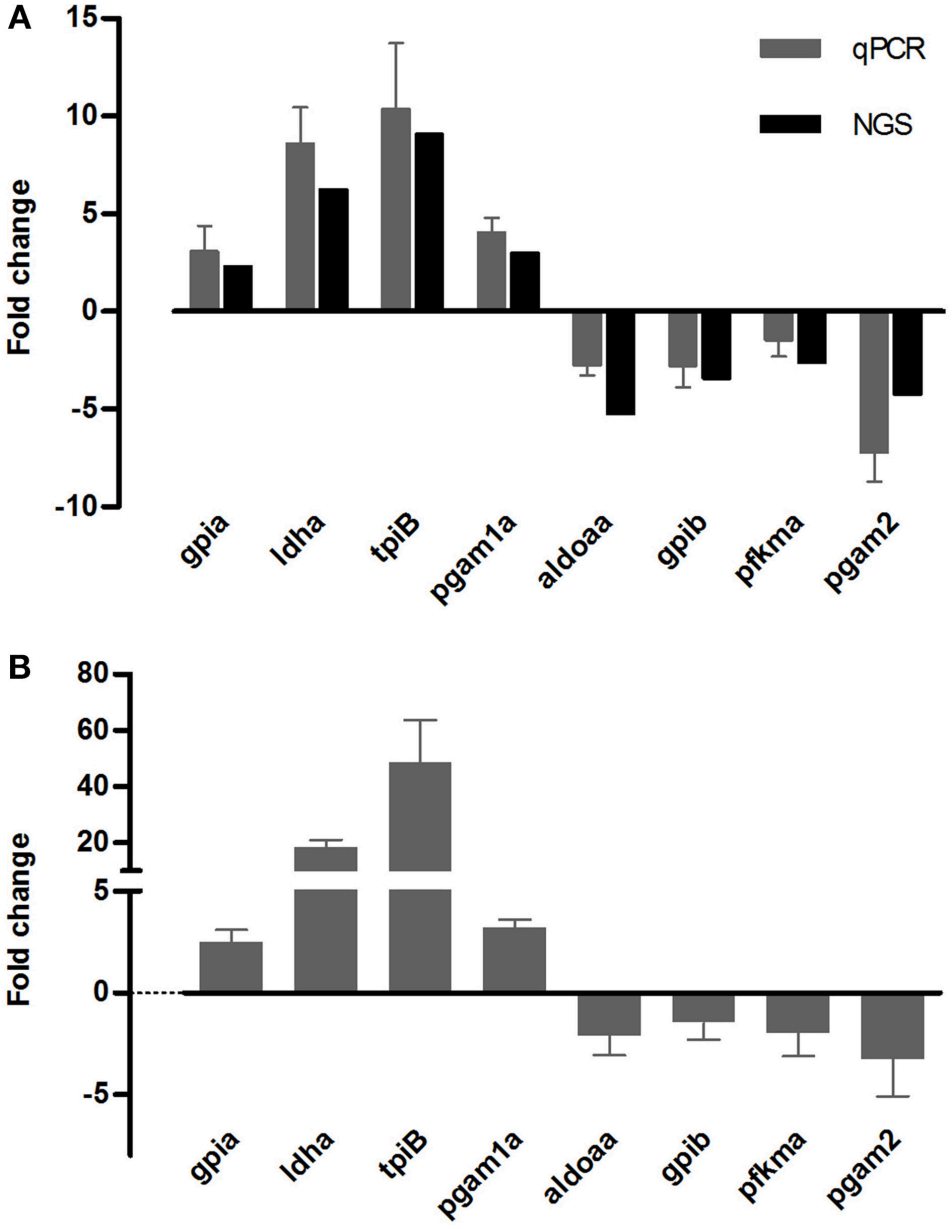
qPCR validation of eight DEG obtained from the Illumina deep-sequencing (NGS) analysis. Comparison of transcript expression levels measured using NGS (black bars), and as calculated from qPCR reactions (gray bars), in the same fish from families 480 and 740 **(A)**. Temperature-dependent expression of the same genes in fish from additional four families (430, 460, 720, 730), analyzed by qPCR **(B)**.

Target genes for qPCR analysis were selected from the genes related to the glycolysis/gluconeogenesis pathway, that had opposite responses between cold tolerant and sensitive fish. Gills samples of nine fish from three tolerant families and nine fish from three sensitive families were analyzed. For all tested genes, the direction of change in expression was concordant between the transcriptome sequencing data and the qPCR analysis (Figure 6). Transcripts levels at 12°C significantly differ from 24°C for *gpia, ldha, tbiB*, and *pgam1a* (*P* < 0.001), for *aldoaa* and *pgam2* (*P* = 0.02), but not for *gpib, pfkma* (*P* = 0.1).

## Discussion

Ectotherm animals, like fish, go through major physiological responses when they acclimate to different temperatures. While the emergence of genomic technologies led to several studies that characterized genes and pathways involved in fish thermal acclimation (Gracey et al., 2004; Long et al., 2012, 2013; Mininni et al., 2014; Hu et al., 2016), there is still considerable lack of knowledge in this area. Moreover, although it is well-known that there is within-species phenotypic variation in thermal tolerance (Tave et al., 1990; Thoa et al., 2014), the physiological basis behind this variation was not investigated so far. In the current study, we used a set-up in which phenotypes were characterized at the family level, enabling comparative transcriptome characterization of fish with the same phenotype, either the tolerant or sensitive, at different temperatures. This system allowed for retrieval of the response to temperature decline at the gene-expression level.

As only one tolerant and one sensitive family were used for the transcriptome sequencing, we examined fish from additional four families, two tolerant and two sensitive, verifying that the observed patterns are universal and the detected differences are not family-specific. By comparing the transcriptional responses to a low-temperature challenge in the gills and liver of cold tolerant and sensitive blue tilapia, we observed the common response between all the fish that were analyzed. Thus, we considered it as the primary response of blue tilapia to low temperature exposure. KEGG analysis of DEG showed down-regulation of genes involved in cellular interactions with the surrounding environment, adjacent cells and the ECM. In contrast, up-regulated genes are involved in intracellular processes, such as protein processing and RNA transport and degradation. These findings are in line with previous transcriptomic analyses that used microarray to study response to low temperatures in fish. Analysis of seven common carp (*Cyprinus carpio*) tissues demonstrated common activation of genes involved in ubiquitin-dependent protein catabolism and proteasome function, a response that was not expected (Gracey et al., 2004). Analysis of gilthead sea bream (*Sparus aurata*) liver transcriptome also revealed broad activation of genes involved in RNA processing, protein catabolism and folding (Mininni et al., 2014). Overall, it can be concluded that at the cellular level, the primary response of fish to low temperature stress is an increase in intracellular processes and decrease in extracellular processes. This might reflect an adjustment process of the cell with a concomitant reduction in the organismal activity.

Most of the variation found in our transcriptome sequences can be attributed to differences between tissues and temperatures. Despite that, our following analyses showed relatively small number of genes that belong to specific pathways are the basis of the difference between tolerant and sensitive fish. The analysis of the transcriptomic response to a decreasing temperature regime highlighted some differences between the cold tolerant and sensitive fish. Sensitive fish showed a wider range of up-regulated genes and pathways than tolerant fish in response to cold exposure, suggesting higher investment of physiological resources by cold-sensitive fish during acclimation to low temperatures. The increase in carbon metabolism observed in the sensitive fish might reflect this higher investment. Furthermore, in their study on common carp, Gracey et al. (2004) found an opposite response of carbohydrate metabolism pathways between tissues of the same fish and assumed a probable shift of energy to organs with higher demands.

In this study, we focused on the gene expression level and identified divergence between the cold-sensitive and cold-tolerant blue tilapia in specific pathways. Two carbon metabolism pathways, glycolysis/gluconeogenesis in the gills and biosynthesis of amino-acids in the liver, were found to be differentially regulated in response to low temperature exposure. These pathways play a key role in supplying the organism's energetic demands. While such reverse expression of these pathways between tolerant and sensitive fish was not known before, we have previously demonstrated that increased expression of the *atp6* gene (mitochondrial ATP synthase) in response to temperature reduction was inversely correlated to the level of cold tolerance (Nitzan et al., 2016). This observation further supports the current results showing that energy related pathways serve as an important component of the cold tolerance trait.

Negative correlation between metabolic rate and tolerance to environmental stress is well-known in farmed animals, with cattle and poultry strains with high temperature tolerance having lower growth rate, as well as other productivity traits (West, 2003; Druyan et al., 2012). However, these are endothermal animals that need to maintain constant body temperature, while in fish, such negative correlation is less intuitive. Several studies on different fish species, described the relationships between metabolism and the response to cold temperatures (Guderley and St-Pierre, 2002; Schulte, 2015), but as far as we know the relationships between metabolism and tolerance of low temperatures has not yet been clearly explained in fish. Our results, showing lower expression of energy-related metabolic pathways in cold tolerant fish, suggest that energy balance has a key role in the fish ability to make the physiological adjustments to the cold environment.

In this work, we used the power of family-phenotype, obtained through selective breeding, in order to characterize the transcriptome response to low temperature exposure, in tolerant and sensitive fish. We revealed pathways that are the core cellular response as well as pathways that differ between cold-tolerant and cold-sensitive fish and might be the basis of within-species variance. Understanding the regulation of these pathways should be a key for the improvement of tilapia's cold tolerance, as ability to control or affect pathways which are the basis of phenotypic variation bear better potential for aquaculture than detection of polymorphism in specific genes, each one with minor effect. This study demonstrates the opportunities in using defined genetic structure for experiments aiming at characterization of the physiology of traits. The presented data can be important for our understanding of this economically important trait in cultured fish, as well as for designing research aiming to improve tilapia's cold tolerance through genetic or physiological manipulations on key pathways.

## Author Contributions

AC conceived and designed the experiments. TZ and AB bred and selected the fish. TS, TN, and AC challenged the fish and sampled tissues. TN performed the RNA analyses. TN, AD-F, JB, FK, and AC analyzed the results. AC and IM secured funding and supervised the project. TN, FK, and AC wrote the manuscript.

### Conflict of Interest Statement

The authors declare that the research was conducted in the absence of any commercial or financial relationships that could be construed as a potential conflict of interest.
