# Supplementary material for: Transcriptome Analysis Reveals Common and Differential Response to Low Temperature Exposure Between Tolerant and Sensitive Blue Tilapia (Oreochromis aureus)
Source: Front Genet. 2019 Feb 26;10:100. doi: 10.3389/fgene.2019.00100 (PMC6399464; doi:10.3389/fgene.2019.00100)
Supplement: Supplementary file 1 [file Table_1.DOCX]

**Table S1.** Sequences of primers used for qPCR.

| **Gene** | **Primer** | **Sequence** |
| --- | --- | --- |
| Aldolase a fructose | aldoaa-F | GGTGGTGGGTATCAAGGTCG |
|  | aldoaa-R | GGCGCAACGCTCATAAAGTC |
| Glucose 6 phophate | gpib-F | CCACACTGGGCCAATAGTCT |
|  | gpib-R | GGAAGTCACAAGGCACCATT |
| Phosphfructokinase muscle a | pfkma-F | GCTCACTTTCCAACCTCTCG |
|  | pfkma-R | CTTCATGATGGGCCTGATTT |
| Phosphofructokinase muscle b | pfkmb-f | TGTGGAACATCTGGTGGAGA |
|  | pfkmb-R | TCTTCCTGCAGTCAAACACG |
| Phosphoglycerate mutase 2 | pgam2-F | GGCTGAAAAGCACGGTGAAG |
|  | pgam2-R | GAGCACGGGCAATGGTATCT |
| Glucose 6 phosphate isomerase | gpia-F | TCAAGAAGCTCACCCCGTTC |
|  | gpia-R | TCGATCTTCTTGGCGAGCTG |
| Lactate dehydrogenase | ldha-F | CAGTGCACAAGATGGTGGTTG |
|  | ldha-R | ATGCATGCCCTGAACAAGTGTA |
| Phosphoglucomutase | pgm1-F | GTGGACACTTTCAAGCCCTTC |
|  | pgm1-R | CACATAAGGACCAACCACTCCAT |
| Triosephosphate isomerase | tpi1b-F | ATCCAGACCCTGAACACTGC |
|  | tpi1b-R | AGCCACCACTGGGAACTATG |
| Beta actin | b actin-F | CCACCCAAAGTTCAGCCATG |
|  | b actin-R | ACGATGGAGGGGAAGACAG |
| Elongation factor | EF1-F | TCAACGCTCAGGTCATCATC |
|  | EF1-R | ACGGTCGATCTTCTCAACCA |
| Phosphoglycerate mutase 1 | pgam1a-F | GACCGACGTTATGCTGACCT |
|  | pgam1a-R | ACATACCCTCCAGGTGCTTG |
| Pyruvate kinase | pkma-F | TGAGATCCCCACAGAAAAGG |
|  | pkma-R | CTACATCACTGCCCTCAGCA |
