# Supplementary material for: Transcriptome Analysis Reveals Common and Differential Response to Low Temperature Exposure Between Tolerant and Sensitive Blue Tilapia (Oreochromis aureus)
Source: Front Genet. 2019 Feb 26;10:100. doi: 10.3389/fgene.2019.00100 (PMC6399464; doi:10.3389/fgene.2019.00100)
Supplement: Supplementary file 2 [file Table_2.DOCX]

**Table S2.** Data of the Illumina sequencing and the mapping to the Nile tilapia reference genome, of the 24 analyzed libraries.

| **Tolerance** | **Temperature** | **Tissue** | **Raw reads** | **Clean reads** | **% of clean reads** | **% of mapping** |
| --- | --- | --- | --- | --- | --- | --- |
| Resistant | 12 | Gills | 34,185,476 | 32,929,254 | 96.3 | 75.6 |
| Resistant | 12 | Gills | 814,322 | 700,410 | 86.0 | 65.4 |
| Resistant | 12 | Gills | 20,640,181 | 18,972,602 | 91.9 | 63.2 |
| Resistant | 12 | Liver | 33,409,959 | 31,873,706 | 95.4 | 66.3 |
| Resistant | 12 | Liver | 18,883,215 | 17,792,851 | 94.2 | 69.1 |
| Resistant | 12 | Liver | 13,215,778 | 4,271,760 | 32.3 | 6.7 |
| Sensitive | 12 | Gills | 23,195,843 | 22,262,386 | 96.0 | 71.9 |
| Sensitive | 12 | Gills | 18,658,350 | 16,586,098 | 88.9 | 57.8 |
| Sensitive | 12 | Gills | 23,122,997 | 22,220,899 | 96.1 | 80.3 |
| Sensitive | 12 | Liver | 30,480,336 | 29,356,926 | 96.3 | 68.0 |
| Sensitive | 12 | Liver | 18,992,107 | 17,662,790 | 93.0 | 72.4 |
| Sensitive | 12 | Liver | 20,581,738 | 16,581,558 | 80.6 | 66.9 |
| Resistant | 24 | Gills | 19,329,733 | 18,207,098 | 94.2 | 70.5 |
| Resistant | 24 | Gills | 17,026,876 | 16,242,644 | 95.4 | 73.4 |
| Resistant | 24 | Gills | 63,995,736 | 44,313,180 | 69.2 | 75.6 |
| Resistant | 24 | Liver | 21,384,504 | 20,592,540 | 96.3 | 74.9 |
| Resistant | 24 | Liver | 15,567,236 | 14,892,073 | 95.7 | 75.0 |
| Resistant | 24 | Liver | 13,161,987 | 12,042,406 | 91.5 | 71.8 |
| Sensitive | 24 | Gills | 19,602,070 | 18,507,727 | 94.4 | 78.7 |
| Sensitive | 24 | Gills | 12,879,862 | 12,072,054 | 93.7 | 70.1 |
| Sensitive | 24 | Gills | 15,633,096 | 14,815,608 | 94.8 | 74.4 |
| Sensitive | 24 | Liver | 16,404,872 | 15,499,596 | 94.5 | 79.2 |
| Sensitive | 24 | Liver | 12,957,265 | 11,866,150 | 91.6 | 52.5 |
| Sensitive | 24 | Liver | 12,720,316 | 11,620,267 | 91.4 | 70.8 |
