# Supplementary material for: Transcriptome Analysis Reveals Common and Differential Response to Low Temperature Exposure Between Tolerant and Sensitive Blue Tilapia (Oreochromis aureus)
Source: Front Genet. 2019 Feb 26;10:100. doi: 10.3389/fgene.2019.00100 (PMC6399464; doi:10.3389/fgene.2019.00100)
Supplement: Supplementary file 3 [file Table_3.DOCX]

| **Table S3A.** Permanova (Adonis) results for transcriptomic response in both the gills and the liver based on Jaccard distances (presence/absence of genes). | | | | | | |
| --- | --- | --- | --- | --- | --- | --- |
|  | d.f. | SS | MS | PseudoF | R^2^ | *P*-value |
| *Main Effects* |  |  |  |  |  |  |
| Tolerance | 1 | 0.0660 | 0.06596 | 0.8491 | 0.01624 | 0.453 |
| Temperature | 1 | 0.3562 | 0.35624 | 4.5859 | 0.08774 | 0.006** |
| Tissue | 1 | 2.0159 | 2.01594 | 25.9513 | 0.49649 | 0.001*** |
|  |  |  |  |  |  |  |
| *Interaction terms* | | | | | | |
| Tolerance x temperature | 1 | 0.0625 | 0.06254 | 0.8051 | 0.01540 | 0.489 |
| Temperature x Tissue | 1 | 0.2778 | 0.27778 | 3.5759 | 0.06841 | 0.022* |
| Tolerance x Tissue | 1 | 0.0678 | 0.06778 | 0.8725 | 0.01669 | 0.438 |
| Residuals | 15 | 1.1652 | 0.07768 |  | 0.28698 |  |
| Total | 22 | 4.0604 |  |  | 1.00000 |  |

*, **Statistical significance at *P* < 0.05 and 0.01, respectively. Permutations n = 999.

d.f., degrees of freedom; SS, sum of squares; MS, mean sum of squares.

| **Table S3B.** Permanova (Adonis) results for transcriptomic response in the gills based on Jaccard distances (presence/absence of genes). | | | | | | |
| --- | --- | --- | --- | --- | --- | --- |
|  | d.f. | SS | MS | PseudoF | R^2^ | *P*-value |
| *Main Effects* |  |  |  |  |  |  |
| Tolerance | 1 | 0.05909 | 0.059086 | 0.8499 | 0.06472 | 0.636 |
| Temperature | 1 | 0.24602 | 0.246016 | 3.5386 | 0.26947 | 0.002** |
|  |  |  |  |  |  |  |
| *Interaction terms* | | | | | | |
| Tolerance x temperature | 1 | 0.05166 | 0.051665 | 0.7431 | 0.05659 | 0.772 |
| Residuals | 8 | 0.55618 | 0.069523 |  | 0.60921 |  |
| Total | 11 | 0.91295 |  |  | 1.00000 |  |

*, **Statistical significance at *P* < 0.05 and 0.01, respectively. Permutations n = 999.

d.f., degrees of freedom; SS, sum of squares; MS, mean sum of squares.

| **Table S3C.** Permanova (Adonis) results for transcriptomic response in the liver based on Jaccard distances (presence/absence of genes). | | | | | | |
| --- | --- | --- | --- | --- | --- | --- |
|  | d.f. | SS | MS | PseudoF | R^2^ | *P*-value |
| *Main Effects* |  |  |  |  |  |  |
| Tolerance | 1 | 0.07472 | 0.07472 | 0.8588 | 0.06658 | 0.506 |
| Temperature | 1 | 0.38282 | 0.38282 | 4.3998 | 0.34108 | 0.005** |
|  |  |  |  |  |  |  |
| *Interaction terms* | | | | | | |
| Tolerance x temperature | 1 | 0.05576 | 0.05576 | 0.6409 | 0.04969 | 0.761 |
| Residuals | 7 | 0.60905 | 0.08701 |  | 0.54265 |  |
| Total | 10 | 1.12235 |  |  | 1.00000 |  |

*, **Statistical significance at *P* < 0.05 and 0.01, respectively. Permutations n = 999.

d.f., degrees of freedom; SS, sum of squares; MS, mean sum of squares.
