# Supplementary material for: Transcriptome Analysis Reveals Common and Differential Response to Low Temperature Exposure Between Tolerant and Sensitive Blue Tilapia (Oreochromis aureus)
Source: Front Genet. 2019 Feb 26;10:100. doi: 10.3389/fgene.2019.00100 (PMC6399464; doi:10.3389/fgene.2019.00100)
Supplement: Supplementary file 4 [file Table_4.docx]

**Table S4.** Temperature-dependent DEG shared between all samples (gills and liver of both tolerant and sensitive fish) highlighted in red for up-regulation and blue for down-regulation.

| **UNIPORT** | **Gene description** | **UNIPORT** | **Gene description** |
| --- | --- | --- | --- |
| B7ZV46 | heat shock 70 kDa protein 1-like (LOC100694403), partial mRNA | F1QV49 | transforming acidic coiled-coil-containing protein 1-like (LOC100690047), transcript variant X2, mRNA |
| A0A0R4I9A8 | heat shock protein 30-like (LOC100692254), mRNA | B9VEN0 | uncharacterized LOC100702170 (LOC100702170), mRNA |
| Q90XG0 | triosephosphate isomerase B-like (LOC100690935), mRNA | B8JLP8 | zinc finger, SWIM-type containing 5 (zswim5), transcript variant X2, mRNA |
| A0A0R4ISA3 | heat shock protein HSP 90-alpha-like (LOC100701982), mRNA | A0A0G2KDR5 | putative golgin subfamily A member 6-like protein 6-like (LOC102083195), mRNA |
| F1Q4Z9 | solute carrier family 35 member F1-like (LOC100700320), mRNA | Q6NWE0 | ester hydrolase C11orf54 homolog (LOC100711217), mRNA |
| Q7ZU46 | heat shock 70 kDa protein 4-like (LOC100690112), transcript variant X2, mRNA | H0WEY0 | NACHT, LRR and PYD domains-containing protein 3-like (LOC100711258), mRNA |
| Q6P5M5 | uncharacterized LOC102076401 (LOC102076401), misc_RNA | X1WBL0 | chromodomain helicase DNA binding protein 3 (chd3), transcript variant X1, mRNA |
| E7F7S8 | rotatin (rttn), mRNA | Q9PVE4 | thyroid hormone receptor beta (tr-beta), transcript variant X5, mRNA |
| A0A0R4ILS8 | hsp70-binding protein 1-like (LOC100694972), mRNA | Q5RI33 | connective tissue growth factor-like (LOC100693059), mRNA |
| E7F9W4 | protein FAM161A-like (LOC100700499), mRNA | A0A0G2KUM0 | RNA-binding protein 5-like (LOC100707900), mRNA |
| Q6DI06 | mitochondrial import inner membrane translocase subunit Tim10-like (LOC100705235), mRNA | Q6GMJ4 | histone deacetylase 11-like (LOC100691456), mRNA |
| E7F7X7 | serine/threonine-protein kinase NIM1-like (LOC100702096), transcript variant X1, mRNA | Q6IQU1 | 3-oxo-5-beta-steroid 4-dehydrogenase-like (LOC100700465), transcript variant 1, mRNA |
| F1Q8W8 | calreticulin-like (LOC100701980), transcript variant X2, mRNA | F1QQZ1 | splicing factor 3B subunit 1-like (LOC100696218), transcript variant X3, mRNA |
| A0A0B5JK63 | calreticulin-like (LOC100701980), transcript variant X1, mRNA | F1Q7R9 | ectonucleoside triphosphate diphosphohydrolase 1-like (LOC100710562), transcript variant X2, mRNA |
| A0A0R4IYB7 | N(alpha)-acetyltransferase 25, NatB auxiliary subunit (naa25), transcript variant X1, mRNA | B3DIZ6 | integrin alpha-IIb-like (LOC100704409), transcript variant X1, mRNA |
| F1QTK7 | dnaJ homolog subfamily A member 1-like (LOC100691603), mRNA | F8W525 | B-cell antigen receptor complex-associated protein alpha chain-like (LOC100698182), mRNA |
| A0A0R4IR32 | bactericidal permeability-increasing protein-like (LOC100694762), mRNA | Q9I9N0 | death-associated protein-like 1-B-like (LOC100707360), transcript variant X1, mRNA |
| B0S595 | carcinoembryonic antigen-related cell adhesion molecule 6-like (LOC100691921), transcript variant X1, mRNA | P31367 | POU domain, class 6, transcription factor 1-like (LOC100712520), transcript variant X3, mRNA |
| Q6NV35 | glutamate-cysteine ligase, catalytic subunit (gclc), transcript variant X1, mRNA | A0A0R4IJ19 | zinc finger protein 628-like (LOC100697888), transcript variant X5, mRNA |
| Q6NZU8 | ATPase family AAA domain-containing protein 3-A-like (LOC100711548), mRNA | F6PBM1 | O-acetyl-ADP-ribose deacetylase MACROD1-like (LOC100703609), transcript variant X2, mRNA |
| B0V3H3 | U1 small nuclear ribonucleoprotein A-like (LOC100697367), transcript variant X5, mRNA | A0A0R4IH59 | actin filament-associated protein 1-like 2-like (LOC100697549), transcript variant X1, mRNA |
| A5WUU0 | dnaJ homolog subfamily B member 5-like (LOC100692197), transcript variant X2, mRNA | B3DJM4 | bone morphogenetic protein 1-like (LOC100704448), transcript variant X1, mRNA |
| Q7SXM1 | cytochrome c oxidase subunit 6B1-like (LOC100697091), mRNA | E7FG27 | ectonucleoside triphosphate diphosphohydrolase 1-like (LOC100710562), transcript variant X1, mRNA |
| E7FD56 | N(alpha)-acetyltransferase 25, NatB auxiliary subunit (naa25), transcript variant X2, mRNA | B0S8K3 | uncharacterized LOC100698387 (LOC100698387), mRNA |
| F1Q7T0 | solute carrier family 35 member F2-like (LOC100696653), mRNA | F1Q4N9 | disintegrin and metalloproteinase domain-containing protein 19-like (LOC100707541), transcript variant X1, mRNA |
| A0A140LH17 | ubiquitin-associated protein 1-like (LOC100707332), mRNA | A0A0G2L4W7 | uncharacterized LOC102080568 (LOC102080568), mRNA |
| F1QSL9 | retinal guanylyl cyclase 2-like (LOC100708131), mRNA | A5Z1Y0 | transcription factor Sp2-like (LOC100702386), transcript variant X2, mRNA |
| B2GRS8 | NADPH-dependent diflavin oxidoreductase 1-like (LOC100690257), transcript variant X1, mRNA | F1QAQ7 | 5-hydroxytryptamine receptor 1D-like (LOC100708195), mRNA |
| Q5XJT8 | glutathione synthetase-like (LOC100706450), partial mRNA | Q6P0S2 | voltage-dependent anion-selective channel protein 2-like (LOC100698339), transcript variant X1, mRNA |
| Q52JI3 | beta-crystallin B3-like (LOC100707280), transcript variant X2, mRNA | A5HLY6 | A-kinase anchor protein 12-like (LOC100699310), transcript variant X1, mRNA |
| F1RDR2 | 28S ribosomal protein S27, mitochondrial-like (LOC100704791), transcript variant X2, mRNA | F2Z4T4 | A-kinase anchor protein 12-like (LOC100699310), transcript variant X4, mRNA |
| B3DFT1 | neuritin-like protein-like (LOC100706636), mRNA | E7FAU0 | midline-1-like (LOC100692412), transcript variant X4, mRNA |
| Q8QFL6 | multidrug resistance-associated protein 4-like (LOC100701348), mRNA | A5YC46 | muscleblind-like protein 1-like (LOC100712433), transcript variant X7, mRNA |
| Q6NWJ4 | dnaJ homolog subfamily C member 2-like (LOC100689704), mRNA | S6BPQ3 | thyroid hormone receptor beta (tr-beta), transcript variant X2, mRNA |
| Q6DHP6 | nuclear prelamin A recognition factor-like (LOC100697918), transcript variant X1, mRNA | Q5RIH2 | c-1-tetrahydrofolate synthase, cytoplasmic-like (LOC102080606), transcript variant X1, mRNA |
| E7F4W0 | peroxisome proliferator-activated receptor gamma coactivator-related protein 1-like (LOC100709586), mRNA | Q19VG7 | anthrax toxin receptor 2-like (LOC100695560), transcript variant X2, mRNA |
| A0A0R4IUP3 | sodium/glucose cotransporter 2-like (LOC100692902), mRNA | A2BGY1 | armadillo repeat protein deleted in velo-cardio-facial syndrome homolog (LOC100707460), transcript variant X2, mRNA |
| E7F131 | transferrin receptor protein 1-like (LOC100702695), transcript variant X2, mRNA | Q08B99 | beta-1,4-galactosyltransferase 1-like (LOC100691259), mRNA |
| B3DHE5 | uncharacterized LOC100698895 (LOC100698895), transcript variant X1, mRNA | B0S6J8 | ankyrin repeat domain-containing protein 10-like (LOC100710526), mRNA |
| A0A0R4IEX1 | centrosomal protein of 192 kDa-like (LOC100702653), transcript variant X3, mRNA | A0A0G2KSN0 | T-cell differentiation antigen CD6-like (LOC100694688), transcript variant X2, mRNA |
| A0A0R4IAU8 | HEAT repeat-containing protein 1-like (LOC100706754), mRNA | A1A5I3 | integrin alpha-L-like (LOC100708437), transcript variant X1, mRNA |
| A0A0R4IJB2 | porphobilinogen deaminase-like (LOC100696491), transcript variant X2, mRNA | B3DKP3 | O-linked N-acetylglucosamine (GlcNAc) transferase (UDP-N-acetylglucosamine:polypeptide-N-acetylglucosaminyl transferase) (ogt), transcript variant X1, mRNA |
| Q4KMJ1 | cation transport regulator-like protein 2-like (LOC100710035), mRNA | B3DIP4 | uncharacterized LOC100692589 (LOC100692589), mRNA |
| F1QA51 | uncharacterized LOC100693956 (LOC100693956), transcript variant X2, mRNA | B5DDZ4 | dipeptidyl peptidase 4-like (LOC100705023), mRNA |
| F1R9Y7 | NADPH-dependent diflavin oxidoreductase 1-like (LOC100690257), transcript variant X3, mRNA | B0R197 | voltage-dependent anion-selective channel protein 2-like (LOC100698339), transcript variant X3, mRNA |
| Q1LVM3 | exosome complex component RRP46-like (LOC100710370), mRNA | Q6EF98 | extracellular sulfatase Sulf-2-like (LOC100707988), transcript variant X1, mRNA |
| Q1LWL7 | tRNA dimethylallyltransferase, mitochondrial-like (LOC100707141), transcript variant X2, mRNA | E9QC14 | C-type lectin domain family 9 member A-like (LOC100696287), transcript variant 1, mRNA |
| F1QZ64 | ATP-binding cassette, sub-family F (GCN20), member 2 (abcf2), transcript variant X2, mRNA | A0A0R4IYQ8 | O-linked N-acetylglucosamine (GlcNAc) transferase (UDP-N-acetylglucosamine:polypeptide-N-acetylglucosaminyl transferase) (ogt), transcript variant X2, mRNA |
| B2GSH6 | mitochondrial import inner membrane translocase subunit Tim13-like (LOC100693054), mRNA | Q1L8U5 | dual specificity protein kinase CLK2-like (LOC100694439), transcript variant X4, mRNA |
| F1QE75 | TBC1 domain family member 4-like (LOC100712298), transcript variant X3, mRNA | F1R9B5 | zinc finger C2HC domain-containing protein 1A-like (LOC100692339), transcript variant X3, mRNA |
| Q6DC18 | 14 kDa phosphohistidine phosphatase-like (LOC100711007), mRNA | E2E3G0 | nuclear factor interleukin-3-regulated protein-like (LOC100707270), mRNA |
| F1QJ77 | geranylgeranyl transferase type-2 subunit alpha-like (LOC100690172), mRNA | F1QEV3 |  |
| A0A0R4ILM9 | URB1 ribosome biogenesis 1 homolog (S. cerevisiae) (urb1), mRNA | A4QN44 | probable ATP-dependent RNA helicase DDX17-like (LOC100692646), mRNA |
| F1Q7U1 | E3 ubiquitin-protein ligase E3D-like (LOC102081776), mRNA | A0A0R4IU97 | poliovirus receptor-related protein 2-like (LOC102080418), mRNA |
| Q6TNS2 | p21-activated protein kinase-interacting protein 1-like (LOC100710247), mRNA | Q4VBI0 | serum paraoxonase/arylesterase 2-like (LOC100710431), mRNA |
| A3KNP4 | uncharacterized LOC100698168 (LOC100698168), mRNA | F1QC71 | anoctamin-5-like (LOC100704073), transcript variant X2, mRNA |
| Q7SZR8 | ribosome biogenesis protein BRX1 homolog (LOC100690314), mRNA | A2CG09 | histone acetyltransferase KAT6A-like (LOC100708172), transcript variant X2, mRNA |
| Q5BKW8 | small integral membrane protein 12-like (LOC100698330), transcript variant X1, mRNA | Q92051 | carbonic anhydrase-like (LOC100709107), mRNA |
| F1RBQ8 | tetratricopeptide repeat protein 25-like (LOC100690988), transcript variant X1, mRNA | Q5RGW3 | T-complex protein 11-like protein 2-like (LOC100701910), mRNA |
| Q568H8 | DNA-directed RNA polymerases I, II, and III subunit RPABC3-like (LOC100711485), transcript variant X2, mRNA | Q7ZSY2 | malate dehydrogenase, cytoplasmic-like (LOC100711978), mRNA |
| Q6NV24 | prostamide/prostaglandin F synthase-like (LOC100710550), transcript variant X1, mRNA | A0A0R4IGV9 | flocculation protein FLO11-like (LOC100691450), transcript variant X3, mRNA |
| F1QGD9 | tRNA (guanine(26)-N(2))-dimethyltransferase-like (LOC100710345), transcript variant X2, mRNA | Q9YHT4 | 5-aminolevulinate synthase, erythroid-specific, mitochondrial-like (LOC100697245), mRNA |
| A8E5E4 | 28S ribosomal protein S34, mitochondrial-like (LOC100697189), mRNA | Q803Y0 | calcineurin B homologous protein 2-like (LOC100695143), transcript variant X1, mRNA |
| F6NHG9 | A-kinase anchor protein 1, mitochondrial-like (LOC100703121), transcript variant X2, mRNA | B8JII5 | RNA exonuclease 1 homolog (LOC100705890), transcript variant X2, mRNA |
| B0R026 | protein arginine N-methyltransferase 5-like (LOC100711244), mRNA | A4JYQ0 | ciliary neurotrophic factor receptor subunit alpha-like (LOC100692782), transcript variant X3, mRNA |
| A8WHP4 | glutamate--cysteine ligase regulatory subunit-like (LOC100708953), transcript variant X1, mRNA | Q7ZU80 | protein lunapark-A-like (LOC100698672), transcript variant X5, mRNA |
| A0A0G2KGY0 | RNA-binding protein with serine-rich domain 1-like (LOC100698415), mRNA | F1R7T3 | GTPase IMAP family member 4-like (LOC102076869), mRNA |
| F1R356 | probable methyltransferase TARBP1-like (LOC100700626), transcript variant X2, mRNA | A0A0G2KV03 | zinc finger protein 135-like (LOC100696676), mRNA |
| G5CTT5 | polyribonucleotide nucleotidyltransferase 1 (pnpt1), mRNA | Q9DGE0 | dual specificity mitogen-activated protein kinase kinase 6-like (LOC100697345), transcript variant X1, mRNA |
| A7YXZ7 | nucleolar protein 7-like (LOC100698539), mRNA | E9QBP8 | forkhead box protein P1-B-like (LOC100707959), transcript variant X4, mRNA |
| F1QDC7 | protein MAK16 homolog (LOC100698088), transcript variant X3, mRNA | A0A0R4IIH4 | FYVE and coiled-coil domain-containing protein 1-like (LOC100690901), transcript variant X6, mRNA |
| A0A0R4IRP7 | mitochondrial import receptor subunit TOM20 homolog (LOC100695809), mRNA | E7F927 | C-type lectin domain family 4 member F-like (LOC102078738), mRNA |
| Q6NZ06 | interleukin enhancer-binding factor 2 homolog (LOC100704823), mRNA | B3DFS9 | myosin-11-like (LOC100691292), mRNA |
| F1QIJ2 | N-alpha-acetyltransferase 35, NatC auxiliary subunit-like (LOC100699474), mRNA | F1QGH6 | APC membrane recruitment protein 2-like (LOC100691528), mRNA |
| A5WV68 | tRNA-splicing endonuclease subunit Sen15-like (LOC100705017), mRNA | F1QTR4 | protein phosphatase 1 regulatory subunit 3C-B-like (LOC100706687), mRNA |
| F1R8Z3 | GPN-loop GTPase 1-like (LOC100692576), transcript variant X2, mRNA | E7F5R0 | bromodomain and PHD finger-containing protein 3-like (LOC100698230), transcript variant X3, mRNA |
| A0A0R4IQS8 | sarcoplasmic/endoplasmic reticulum calcium ATPase 2-like (LOC100710397), transcript variant X1, mRNA | E7FAF7 | circularly permutated Ras protein 1-like (LOC100692219), mRNA |
| F8W3P6 | protein MAK16 homolog (LOC100698088), transcript variant X1, mRNA | A5YC47 | muscleblind-like protein 1-like (LOC100712433), transcript variant X1, mRNA |
| Q7SX91 | ribosome biogenesis regulatory protein homolog (LOC100703703), mRNA | Q6NYQ0 | tropomyosin alpha-4 chain-like (LOC100711543), transcript variant X4, mRNA |
| B0UXP9 | IMP (inosine 5'-monophosphate) dehydrogenase 2 (impdh2), transcript variant X4, mRNA | X1WDW0 | putative uncharacterized protein C1orf210-like (LOC100693370), mRNA |
| B8A5D4 | thioredoxin-like protein 1-like (LOC100702070), transcript variant X1, mRNA | Q2ABP1 | bone morphogenetic protein 1-like (LOC100704448), transcript variant X2, mRNA |
| G1K2X8 | exportin-T-like (LOC100695406), transcript variant X2, mRNA | F1Q529 | thyroid hormone receptor interactor 12 (trip12), transcript variant X7, mRNA |
| Q05AN2 | 39S ribosomal protein L21, mitochondrial-like (LOC100710875), mRNA | Q1LUU8 | zinc finger protein 384-like (LOC100704458), transcript variant X1, mRNA |
| B8JKQ1 | ESF1 homolog (LOC100693913), transcript variant X1, mRNA | E7F188 | uncharacterized LOC100710458 (LOC100710458), transcript variant X1, mRNA |
| Q5TYS0 | lactation elevated protein 1 homolog B-like (LOC100696370), mRNA | Q32ZE7 | aquaporin-1-like (LOC100692669), mRNA |
| B5DDH6 | elongator complex protein 2-like (LOC100695472), mRNA | Q1L8U4 | dual specificity protein kinase CLK2-like (LOC100694439), transcript variant X1, mRNA |
| Q6DG36 | zinc transporter 5-like (LOC100706897), transcript variant X2, mRNA | A0A0G2KYX9 | general transcription factor II-I repeat domain-containing protein 2-like (LOC102076264), mRNA |
| Q7ZV77 | proteasome subunit alpha type-4-like (LOC100700300), transcript variant X2, mRNA | E7FAE1 | zinc finger protein 618-like (LOC100697547), mRNA |
| Q498Z9 | dyskeratosis congenita 1, dyskerin (dkc1), transcript variant X2, mRNA | A0A140LG26 | equilibrative nucleoside transporter 1-like (LOC100704394), transcript variant X1, mRNA |
| Q5BJJ8 | 39S ribosomal protein L51, mitochondrial-like (LOC100711314), mRNA | A9UH06 | muscleblind-like protein 1-like (LOC100712433), transcript variant X12, mRNA |
| F1QTY5 | tRNA (adenine(58)-N(1))-methyltransferase catalytic subunit TRMT61A-like (LOC100696453), transcript variant X4, mRNA | Q5SPG8 | acyl-coenzyme A thioesterase 3-like (LOC100693079), transcript variant X2, mRNA |
| Q6IQL7 | peptidyl-prolyl cis-trans isomerase D-like (LOC100691620), mRNA | E7F8U8 | cytokine-dependent hematopoietic cell linker-like (LOC100706919), transcript variant X3, mRNA |
| E7F784 | ATP-binding cassette sub-family F member 3-like (LOC100694648), transcript variant X2, mRNA | F1RDW1 | pre-B-cell leukemia transcription factor-interacting protein 1-like (LOC100709266), transcript variant X1, mRNA |
| F1RAE2 | H/ACA ribonucleoprotein complex subunit 1-like (LOC100705748), mRNA | A0A0G2KLK7 | zinc finger and SCAN domain-containing protein 2-like (LOC100695786), transcript variant X4, mRNA |
| Q6TH20 | developmentally regulated GTP binding protein 2 (drg2), transcript variant X1, mRNA | E7EDQ9 | SPARC-related modular calcium-binding protein 1-like (LOC100711651), transcript variant X3, mRNA |
| E7EZJ0 | nucleolar protein 9-like (LOC102076389), transcript variant X2, mRNA | F1QLY4 | nuclear receptor ROR-alpha-like (LOC100689995), transcript variant X2, mRNA |
| Q8JHJ2 | ATP-dependent RNA helicase DDX55-like (LOC100701952), mRNA | F1Q752 | kielin/chordin-like protein-like (LOC100707160), mRNA |
| F1QT47 | gamma-tubulin complex component 2-like (LOC100699933), transcript variant X1, mRNA | F1QJB9 | E3 ubiquitin/ISG15 ligase TRIM25-like (LOC100703139), mRNA |
| Q32LR6 | glioma tumor suppressor candidate region gene 2 protein-like (LOC100691016), mRNA | A0A0R4IUY8 | histone-lysine N-methyltransferase PRDM9-like (LOC100709046), transcript variant X1, mRNA |
| Q6DRL3 | WD repeat domain 46 (wdr46), mRNA | A2BIN4 | splicing regulatory glutamine/lysine-rich protein 1-like (LOC100695937), transcript variant X4, mRNA |
| Q32PN9 | guanine nucleotide-binding protein-like 3-like (LOC100691609), mRNA | E7FAK2 | spindlin-1-like (LOC100700539), transcript variant X3, mRNA |
| B7ZDB0 | uncharacterized LOC102081165 (LOC102081165), mRNA | A2RUY1 | large neutral amino acids transporter small subunit 3-like (LOC100703499), mRNA |
| Q08CQ9 | nucleolin-like (LOC100701691), transcript variant X2, mRNA | A0A0R4INQ0 | LIM and calponin homology domains-containing protein 1-like (LOC100711287), transcript variant X7, mRNA |
| Q7ZV23 | NHP2-like protein 1-like (LOC100708852), mRNA | Q90YJ1 | collagen, type I, alpha 1 (col1a1), transcript variant X1, mRNA |
| C6EVZ7 | hydroxycarboxylic acid receptor 3-like (LOC100690406), mRNA | F1R394 | zinc finger protein 438-like (LOC100705344), transcript variant X3, mRNA |
| A7MCI3 | high affinity copper uptake protein 1-like (LOC100692745), transcript variant X3, mRNA | F1QVS2 | uncharacterized LOC100699521 (LOC100699521), mRNA |
| Q4V9K8 | DEAD (Asp-Glu-Ala-Asp) box polypeptide 18 (ddx18), mRNA | E7FF10 | von Willebrand factor A domain-containing protein 1-like (LOC100702279), mRNA |
| E7FCP9 | TNFAIP3-interacting protein 1-like (LOC100710289), mRNA | F1R677 | patatin-like phospholipase domain-containing protein 7-like (LOC100698638), transcript variant X3, mRNA |
| Q6DHI9 | proteasome subunit beta type-2-like (LOC100710147), transcript variant X1, mRNA | B8JKG7 | patatin-like phospholipase domain-containing protein 4-like (LOC100696211), transcript variant X4, mRNA |
| A0A0G2L325 | ribosomal RNA processing protein 1 homolog B-like (LOC100690727), mRNA | A0A0G2L3U3 | nucleus accumbens-associated protein 1-like (LOC100693985), transcript variant X1, mRNA |
| F1R3Q7 | U3 small nucleolar RNA-interacting protein 2-like (LOC100708981), transcript variant X1, mRNA | F1QBM2 | programmed cell death protein 4-like (LOC100708189), mRNA |
| Q4VBT6 | exosome complex component RRP43-like (LOC100706877), mRNA | F1QDB2 | thrombopoietin receptor-like (LOC100693899), mRNA |
| F1Q9R9 | potassium/sodium hyperpolarization-activated cyclic nucleotide-gated channel 2-like (LOC100706079), mRNA | E7F6D4 | glucosidase, alpha; neutral C (ganc), transcript variant X1, mRNA |
| Q5BLH8 | mitochondrial import inner membrane translocase subunit TIM44-like (LOC100692578), mRNA | E9QB46 | selenium-binding protein 1-like (LOC100691953), mRNA |
| A0A0R4IA19 | DEAD (Asp-Glu-Ala-Asp) box polypeptide 24 (ddx24), mRNA | Q6NY60 | intermediate filament protein ON3-like (LOC100696601), mRNA |
| F1QHE5 | proteasome (prosome, macropain) 26S subunit, non-ATPase, 14 (psmd14), mRNA | E7F5I3 | periostin-like (LOC100695303), transcript variant X2, mRNA |
| A9JRI0 | HEAT repeat-containing protein 6-like (LOC100704793), mRNA | Q6IQ71 | chromobox protein homolog 1-like (LOC100704261), transcript variant X2, mRNA |
| Q6P3I0 | HEAT repeat containing 3 (heatr3), mRNA | Q1L9F1 | keratinocyte-associated protein 3-like (LOC100702712), mRNA |
| Q5U3Q5 | transducin beta-like protein 3-like (LOC100707920), transcript variant X1, mRNA | B0CLW6 | poliovirus receptor-related protein 2-like (LOC100707658), transcript variant X2, mRNA |
| Q5BLA5 | nucleoplasmin-like protein NO29-like (LOC100690530), mRNA | Q6PBT6 | GTP cyclohydrolase 1 feedback regulatory protein-like (LOC100707287), mRNA |
| X1WET4 | putative ATP-dependent RNA helicase DHX33-like (LOC100690962), transcript variant X1, mRNA | Q6YLV7 | Krueppel-like factor 15-like (LOC100690110), mRNA |
| P79741 | pescadillo-like (LOC100712031), transcript variant X3, mRNA | A0A0R4IA85 | poliovirus receptor homolog (LOC102079940), mRNA |
| B2GQM3 | nucleolar protein 10-like (LOC100690372), transcript variant X1, mRNA | F1RCC9 | synaptopodin-2-like (LOC100708639), transcript variant X4, mRNA |
| G1K2I9 | methyltransferase-like protein 13-like (LOC100708771), mRNA | F1R0R6 | prostaglandin G/H synthase 1-like (LOC100699955), mRNA |
| Q6AZC1 | 26S protease regulatory subunit 8-like (LOC100690634), transcript variant X1, mRNA | Q7T372 | actin-related protein 2/3 complex subunit 1B-like (LOC100695691), mRNA |
| A1L243 | LDLR chaperone MESD-like (LOC102080808), mRNA | F1RAF7 | RNA polymerase II elongation factor ELL-like (LOC100703490), mRNA |
| Q6DGY8 | proteasome subunit alpha type-6-like (LOC100691978), mRNA | F1QPV1 | myosin light chain kinase, smooth muscle-like (LOC100690170), partial mRNA |
| Q568H9 | rRNA-processing protein FCF1 homolog (LOC100694422), transcript variant X1, mRNA | A9JSY0 | atlastin-2-like (LOC100695949), transcript variant X3, misc_RNA |
| Q7SZD2 | 28 kDa heat- and acid-stable phosphoprotein-like (LOC100698452), transcript variant X2, mRNA | F1Q6L3 | forkhead box protein N2-like (LOC100703598), transcript variant X2, mRNA |
| A0A0R4III8 | G protein-regulated inducer of neurite outgrowth 2-like (LOC102081675), transcript variant X2, mRNA | E7F1U0 | uncharacterized LOC100707877 (LOC100707877), mRNA |
| A0A0R4IB93 | ubiquitin specific peptidase 48 (usp48), mRNA | Q5RI27 | scavenger receptor cysteine-rich type 1 protein M160-like (LOC102076712), mRNA |
| Q4V8Q4 | vesicle transport protein GOT1B-like (LOC100702173), transcript variant X2, mRNA | Q6AXK6 | protein yippee-like 1-like (LOC100701275), transcript variant X2, mRNA |
| X1WF30 | tRNA (adenine(58)-N(1))-methyltransferase, mitochondrial-like (LOC100710847), transcript variant X3, mRNA | Z4YI19 | tyrosyl-DNA phosphodiesterase 2-like (LOC100706541), mRNA |
| A0A0R4IPY9 | collagenase 3-like (LOC100711125), mRNA | Q7M561 | DNA repair and recombination protein pif1, mitochondrial-like (LOC102079047), mRNA |
| B2GSP4 | NEDD8-activating enzyme E1 catalytic subunit-like (LOC100698283), transcript variant X1, mRNA | Q0P497 | Krueppel-like factor 11-like (LOC100691904), transcript variant X2, mRNA |
| Q6DGH5 | ankyrin repeat and SOCS box protein 13-like (LOC100709988), transcript variant X1, mRNA | A0A0R4IZN5 | CMRF35-like molecule 3-like (LOC100697751), mRNA |
| P83571 | ruvB-like 2-like (LOC100708215), transcript variant X1, mRNA | F1QZ17 | pleckstrin homology domain-containing family H member 1-like (LOC100695820), mRNA |
| Q66I02 | something about silencing protein 10-like (LOC100699931), mRNA | Q7T3A2 | uncharacterized LOC102076770 (LOC102076770), mRNA |
| Q5PR27 | 39S ribosomal protein L1, mitochondrial-like (LOC100698311), mRNA | Q1XHK0 | glucocorticoid receptor 2b (gr2b), transcript variant X2, mRNA |
| Q7T314 | mitochondrial import receptor subunit TOM40 homolog (LOC100696843), transcript variant X1, mRNA | Q561U7 | ankyrin repeat and SOCS box protein 9-like (LOC100709637), transcript variant X1, mRNA |
| B2GTD1 | ribosome biogenesis protein wdr12-like (LOC100712521), mRNA | A2VCY4 | very long-chain acyl-CoA synthetase-like (LOC100710099), mRNA |
| Q6T9C2 | regulator of G-protein signaling 12-like (LOC100696390), transcript variant X2, mRNA | B3DKL4 | cholinesterase-like (LOC100692786), mRNA |
| A7E2N8 | kxDL motif-containing protein 1-like (LOC100694034), mRNA | F1Q924 | collagen alpha-4(VI) chain-like (LOC102079030), mRNA |
| Q6ZM32 | ADP-ribosylation factor-like protein 5B-like (LOC100708858), mRNA | F6NJS4 | atlastin-2-like (LOC100695949), transcript variant X4, misc_RNA |
| Q6DHU2 | RNA-binding protein 34-like (LOC100698176), mRNA | Q90YJ0 | collagen alpha-2(I) chain-like (LOC100710094), transcript variant X3, mRNA |
| Q6DGV7 | mpv17-like protein 2-like (LOC100694515), transcript variant X2, mRNA | A0A140LH61 | synaptopodin-2-like (LOC100708639), transcript variant X2, mRNA |
| Q6AXK9 | THO complex subunit 3-like (LOC100692987), mRNA | Q5U3T8 | rho-related GTP-binding protein RhoU-like (LOC100696298), mRNA |
| E7EXW2 | phosphatidylinositol glycan anchor biosynthesis, class N (pign), transcript variant X1, mRNA | Q802G7 | selenoprotein M-like (LOC100701119), misc_RNA |
| A0A0R4IGM5 | U3 small nucleolar RNA-associated protein 6 homolog (LOC100702188), mRNA | E7F408 | UDP-glucuronosyltransferase 2B10-like (LOC100700159), mRNA |
| Q7SZR9 | methylosome protein 50-like (LOC100692961), mRNA | F1QNT3 | tubulin-specific chaperone cofactor E-like protein-like (LOC100708677), transcript variant X7, mRNA |
| Q6PC76 | Golgi reassembly-stacking protein 2-like (LOC100696802), transcript variant X2, mRNA | E9QCI3 | synaptopodin-2-like (LOC100708639), transcript variant X3, mRNA |
| Q7ZUT9 | DEAD (Asp-Glu-Ala-Asp) box polypeptide 49 (ddx49), mRNA | F1RD24 | transmembrane and tetratricopeptide repeat containing 2 (tmtc2), transcript variant X1, mRNA |
| A0A0R4ICX1 | solute carrier family 13 (sodium/sulfate symporters), member 4 (slc13a4), mRNA | Q568E5 | vasodilator-stimulated phosphoprotein-like (LOC100692448), mRNA |
| Q7SXM8 | putative methyltransferase NSUN5-like (LOC100690014), mRNA | E7FCX5 | dematin-like (LOC102075582), transcript variant X1, mRNA |
| Q7ZYX7 | 26S proteasome non-ATPase regulatory subunit 7-like (LOC100698291), mRNA | Q5PNP1 | sorting nexin-14-like (LOC100711138), transcript variant X1, mRNA |
| Q6DGX8 | proteasome subunit alpha type-1-like (LOC100703299), mRNA | E9QDD3 | UDP-glucuronosyltransferase 2A3-like (LOC100695035), mRNA |
| Q08BC7 | transmembrane and coiled-coil domains protein 1-like (LOC100692505), transcript variant X6, mRNA | B0R1D5 | polycomb protein suz12-like (LOC100701898), mRNA |
| Q6IQ68 | E3 ubiquitin-protein ligase RNF14-like (LOC100706342), mRNA | A0A0R4IVQ8 | high affinity immunoglobulin gamma Fc receptor I-like (LOC102082917), mRNA |
| F1QTC6 | 2-oxoglutarate and iron-dependent oxygenase domain-containing protein 1-like (LOC100708245), mRNA | A0A0R4IUZ1 | dematin-like (LOC100701040), mRNA |
| F1QGS3 | speckle targeted PIP5K1A-regulated poly(A) polymerase-like (LOC100689816), mRNA | F1QKS1 | cyclin-dependent kinase 19-like (LOC100691935), transcript variant X4, misc_RNA |
| Q6DEG5 | RNA polymerase II (LOC100534454), mRNA | A3KGZ3 | xylose isomerase-like (LOC100703647), transcript variant X2, mRNA |
| Q6NWK4 | V-type proton ATPase subunit E 1-like (LOC100708725), transcript variant X1, mRNA | F1QLR1 | cytochrome c oxidase subunit 4 isoform 2, mitochondrial-like (LOC100704650), mRNA |
| R4GDR2 | U2 small nuclear ribonucleoprotein B''-like (LOC100711151), mRNA | F1QBZ9 | multiple epidermal growth factor-like domains protein 8-like (LOC102081967), mRNA |
| E7FCD8 | V-type proton ATPase catalytic subunit A-like (LOC100710754), mRNA | F1QIE2 | MARVEL domain-containing protein 2-like (LOC100709806), mRNA |
| Q803C2 | transmembrane protein 33-like (LOC100693427), transcript variant X2, mRNA | Q29YB2 | zinc finger protein PLAG1-like (LOC100695438), mRNA |
| F1RCM3 | proteasome subunit beta type-6-like (LOC100694980), mRNA | Q5TYP3 | 14 kDa phosphohistidine phosphatase-like (LOC100707253), mRNA |
| Q6DI25 | U3 small nucleolar ribonucleoprotein protein IMP4-like (LOC100698107), mRNA | A0A0R4IKX6 | Ig heavy chain Mem5-like (LOC100704660), mRNA |
| B0UYL5 | dehydrogenase/reductase SDR family member 12-like (LOC100700592), mRNA | Q6IQN8 | long-chain specific acyl-CoA dehydrogenase, mitochondrial-like (LOC100698696), mRNA |
| A0A0R4IQW7 | UTP20, small subunit (SSU) processome component, homolog (yeast) (utp20), transcript variant X1, mRNA | E7EZ94 | glutathione S-transferase theta-1-like (LOC100696000), mRNA |
| B0JZP5 | deoxyribonuclease-2-alpha-like (LOC100700356), mRNA | Q66I20 | pigment epithelium-derived factor-like (LOC100690960), transcript variant X2, mRNA |
| Q5RIF1 | leukotriene A-4 hydrolase-like (LOC100705920), partial mRNA | Q6NVA6 | myosin regulatory light polypeptide 9-like (LOC100697131), mRNA |
| E7F8W7 | FAST kinase domain-containing protein 1-like (LOC100704428), transcript variant X2, mRNA | B3DIE5 | gamma-aminobutyric acid receptor subunit rho-3-like (LOC100708037), mRNA |
| F1QHE2 | zinc phosphodiesterase ELAC protein 2-like (LOC100705666), transcript variant X3, mRNA | Q6P0S5 | alcohol dehydrogenase 1-like (LOC100702802), mRNA |
| F1R300 | U2 small nuclear ribonucleoprotein A'-like (LOC100697117), mRNA | Q6VN46 |  |
| Q1ECW4 | DEAH (Asp-Glu-Ala-His) box polypeptide 35 (dhx35), mRNA | F1Q7N1 | vitrin (vit), transcript variant X4, mRNA |
| Q6DRE7 | probable ATP-dependent RNA helicase DDX56-like (LOC100702513), mRNA | Q6NYL3 | peroxisomal bifunctional enzyme-like (LOC100709434), mRNA |
| A9JRX9 | nitric oxide-associated protein 1-like (LOC100693515), mRNA | A0A0H2UKX4 | E3 ubiquitin-protein ligase parkin-like (LOC100708029), transcript variant X2, mRNA |
| Q6L5P4 | 28S ribosomal protein S33, mitochondrial-like (LOC100695777), transcript variant X2, mRNA | A0A0R4J7E0 | uncharacterized LOC100699848 (LOC100699848), mRNA |
| Q6TGV4 | 26S proteasome non-ATPase regulatory subunit 13-like (LOC100690318), mRNA | Q98T96 | cytochrome P450 2J2-like (LOC100701527), mRNA |
| F1QQ17 | ribosomal RNA small subunit methyltransferase NEP1-like (LOC100709353), transcript variant 1, mRNA | A0A0R4IIN1 | granzyme G-like (LOC100692779), mRNA |
| F8W4C4 | ubiquitin specific peptidase 5 (isopeptidase T) (usp5), transcript variant X5, mRNA |  |  |
| B8JIY2 | nucleolar and coiled-body phosphoprotein 1-like (LOC100707070), transcript variant X1, mRNA |  |  |
| Q6PGV5 | poly(A) polymerase gamma-like (LOC102082704), transcript variant X1, mRNA |  |  |
| Q7SZC5 | nucleoporin NDC1-like (LOC100701468), transcript variant X2, mRNA |  |  |
| Q0P4A8 | G patch domain-containing protein 4-like (LOC100696289), transcript variant X1, mRNA |  |  |
| F1Q5L6 | probable ATP-dependent RNA helicase DDX5-like (LOC100712452), transcript variant X1, mRNA |  |  |
| E7FA12 | trafficking protein particle complex 8 (trappc8), transcript variant X2, mRNA |  |  |
| Q6IQM2 | cytochrome c-like (LOC100692041), transcript variant X2, mRNA |  |  |
| Q6DBY0 | nuclear pore complex protein Nup85-like (LOC100691023), transcript variant X1, mRNA |  |  |
| F1QH23 | THUMP domain-containing protein 3-like (LOC100693768), transcript variant X2, mRNA |  |  |
| F1Q4S6 | nucleolar protein 6-like (LOC100708207), mRNA |  |  |
| X1WGE9 | DNA-directed RNA polymerase I subunit RPA34-like (LOC100704830), transcript variant X2, mRNA |  |  |
| F1R3J9 | protein arginine N-methyltransferase 1-like (LOC100700512), transcript variant X2, mRNA |  |  |
| A1A5V7 | transmembrane protein 41B-like (LOC100692333), mRNA |  |  |
| F8W4V6 | G-rich sequence factor 1-like (LOC100701818), transcript variant X2, mRNA |  |  |
| F1QR86 | pre-rRNA-processing protein TSR2 homolog (LOC100700686), mRNA |  |  |
| F1QWP7 | putative ribosomal RNA methyltransferase NOP2-like (LOC100709797), mRNA |  |  |
| E7F5L8 | U3 small nucleolar ribonucleoprotein protein MPP10-like (LOC100692797), transcript variant X1, mRNA |  |  |
| A0A0R4II42 | protein CCSMST1-like (LOC100697653), transcript variant 1, mRNA |  |  |
| E7FH60 | uncharacterized LOC100697116 (LOC100697116), mRNA |  |  |
| A0A0R4IFI0 | regulatory-associated protein of mTOR-like (LOC100703173), transcript variant X5, mRNA |  |  |
| A0A0R4IQB8 | pre-rRNA processing protein FTSJ3-like (LOC100696778), mRNA |  |  |
| Q1LVL5 | mitochondrial folate transporter/carrier-like (LOC100702034), mRNA |  |  |
| Q7ZWE1 | periodic tryptophan protein 1 homolog (LOC100700277), mRNA |  |  |
| Q7SXN5 | dynamin-1-like protein-like (LOC100699313), transcript variant X1, mRNA |  |  |
| E7F2M6 | tumor suppressor p53-binding protein 1-like (LOC100711423), transcript variant X1, mRNA |  |  |
| F1QAD8 | dol-P-Man:Man(5)GlcNAc(2)-PP-Dol alpha-1,3-mannosyltransferase-like (LOC100699348), mRNA |  |  |
| F1QPP5 | PCI domain containing 2 (pcid2), mRNA |  |  |
| B2GR20 | 60S ribosome subunit biogenesis protein NIP7 homolog (LOC100693104), transcript variant X1, mRNA |  |  |
| F1QNV4 | nucleoporin 133kDa (nup133), transcript variant X2, mRNA |  |  |
| F1QNA0 | presequence protease, mitochondrial-like (LOC100690836), mRNA |  |  |
| Q7ZU99 | transitional endoplasmic reticulum ATPase-like (LOC100691928), mRNA |  |  |
| Q4V918 | proteasome subunit alpha type-3-like (LOC100694719), mRNA |  |  |
| O42364 | apolipoprotein Eb-like (LOC100699941), mRNA |  |  |
| F5H8N4 | leucine-rich PPR motif-containing protein, mitochondrial-like (LOC100694568), mRNA |  |  |
| A0A0R4IRK6 | exosome complex component RRP40-like (LOC100699556), mRNA |  |  |
| Q4KMF4 | RRP12-like protein-like (LOC100698391), mRNA |  |  |
| A8E5B2 | protein FAM207A-like (LOC100703035), transcript variant 2, mRNA |  |  |
| Q6AZB5 | stromal cell-derived factor 2-like protein 1-like (LOC100696315), mRNA |  |  |
| F1RA21 | 39S ribosomal protein L27, mitochondrial-like (LOC100710199), mRNA |  |  |
| A0A0R4ICQ6 | 26S proteasome non-ATPase regulatory subunit 8-like (LOC100707147), mRNA |  |  |
| B3DJT3 | ataxin-10-like (LOC100702740), mRNA |  |  |
| E7F349 | BMS1 homolog, ribosome assembly protein (yeast) (bms1), mRNA |  |  |
| F1QJP9 | RNA exonuclease 4-like (LOC100700793), mRNA |  |  |
| Q6NZ07 | nicalin-1-like (LOC100707254), transcript variant X2, mRNA |  |  |
| F1QMY8 | RING finger protein 223-like (LOC102079373), transcript variant X2, mRNA |  |  |
| A0A0R4IYN8 | protein SDA1 homolog (LOC100710789), transcript variant X1, mRNA |  |  |
| B8JM36 | 39S ribosomal protein L16, mitochondrial-like (LOC100696981), mRNA |  |  |
| Q6PBK1 | coiled-coil domain-containing protein 58-like (LOC100697851), transcript variant X1, mRNA |  |  |
| Q7T3F9 | 39S ribosomal protein L39, mitochondrial-like (LOC100695635), mRNA |  |  |
| Q7ZU96 | T-complex protein 1 subunit theta-like (LOC100704076), transcript variant X1, mRNA |  |  |
| Q5U3G6 | protein YIF1B-like (LOC100697325), mRNA |  |  |
| F1QAJ4 | glutamyl-tRNA(Gln) amidotransferase subunit A, mitochondrial-like (LOC100695455), mRNA |  |  |
| Q5D018 | RNA-binding protein 8A-like (LOC100703520), transcript variant X1, mRNA |  |  |
| Q6GMK2 | bifunctional methylenetetrahydrofolate dehydrogenase/cyclohydrolase, mitochondrial-like (LOC100702918), mRNA |  |  |
| A2CG10 | adaptor-related protein complex 3, mu 2 subunit (ap3m2), mRNA |  |  |
| F1R498 | RNA polymerase II-associated protein 3-like (LOC100710493), transcript variant X2, mRNA |  |  |
| Q6P6E0 | ATP synthase subunit g, mitochondrial-like (LOC100709019), mRNA |  |  |
| Q7ZTY5 | translation initiation factor eIF-2B subunit gamma-like (LOC100709571), mRNA |  |  |
| Q9IA81 | coilin-like (LOC100711383), mRNA |  |  |
| A0A0R4IN79 | nuclear factor NF-kappa-B p105 subunit-like (LOC100702187), mRNA |  |  |
| A1L1V1 | protein farnesyltransferase/geranylgeranyltransferase type-1 subunit alpha-like (LOC100705878), transcript variant X2, mRNA |  |  |
| E9QH84 | gem-associated protein 5-like (LOC100693103), transcript variant X1, mRNA |  |  |
| Q6P3L3 | 78 kDa glucose-regulated protein-like (LOC100706688), transcript variant X1, mRNA |  |  |
| Q6P0I2 | proteasome subunit alpha type-7-like (LOC100702859), transcript variant 1, mRNA |  |  |
| B0R1D0 | proteasome (prosome, macropain) 26S subunit, ATPase, 4 (psmc4), mRNA |  |  |
| A0A0R4IKX4 | pre-rRNA-processing protein TSR1 homolog (LOC100694129), transcript variant X1, mRNA |  |  |
| Q1L8H2 | mTERF domain-containing protein 1, mitochondrial-like (LOC100705218), transcript variant X1, mRNA |  |  |
| G3N3Y4 | ribosome biogenesis protein bop1-like (LOC100693046), mRNA |  |  |
| A2CEY8 | U3 small nucleolar RNA-associated protein 14 homolog A-like (LOC100701316), mRNA |  |  |
| Q1MT86 | ubiquitin specific peptidase 5 (isopeptidase T) (usp5), transcript variant X3, mRNA |  |  |
| X1WBJ0 | tripartite motif-containing protein 65-like (LOC100692313), mRNA |  |  |
| Q6IQT8 | ruvB-like 1-like (LOC100697768), mRNA |  |  |
| A8HAL1 | ubiquitin carboxyl-terminal hydrolase 16-like (LOC100695149), transcript variant X2, mRNA |  |  |
| Q803D9 | protein arginine N-methyltransferase 1-like (LOC100700512), transcript variant X1, mRNA |  |  |
| Q5RHZ5 | uncharacterized LOC100693539 (LOC100693539), transcript variant X2, mRNA |  |  |
| F1R4C1 | RNA-binding protein NOB1-like (LOC100712241), transcript variant X3, mRNA |  |  |
| Q6DH88 | cytochrome c oxidase protein 20 homolog (LOC100693926), mRNA |  |  |
| A0A0B5JF91 | mesencephalic astrocyte-derived neurotrophic factor-like (LOC100704559), mRNA |  |  |
| Q7SXP2 | NEDD8-activating enzyme E1 regulatory subunit-like (LOC100695127), transcript variant X2, mRNA |  |  |
| Q1LV08 | nuclear pore complex protein Nup93-like (LOC100696986), transcript variant X3, mRNA |  |  |
| Q0P410 | probable methyltransferase BTM2 homolog (LOC100711560), transcript variant X1, mRNA |  |  |
| F1QTR9 | DNA-(apurinic or apyrimidinic site) lyase-like (LOC100699309), mRNA |  |  |
| B8A5U4 | target of EGR1 protein 1-like (LOC100692836), mRNA |  |  |
| Q7SZQ8 | prostaglandin E synthase 3-like (LOC100706085), mRNA |  |  |
| J3JS14 | nucleoporin Nup37-like (LOC100702729), transcript variant X1, mRNA |  |  |
| Q6IQC6 | 26S proteasome non-ATPase regulatory subunit 4-like (LOC102080581), mRNA |  |  |
| F1RBM2 | protein FAM86A-like (LOC100699463), mRNA |  |  |
| Q08C57 | adrenodoxin-like protein, mitochondrial-like (LOC100699751), mRNA |  |  |
| Q6DG91 | proteasome assembly chaperone 1-like (LOC100706404), transcript variant X1, mRNA |  |  |
| Q1LWK8 | major facilitator superfamily domain-containing protein 12-like (LOC100701692), transcript variant X2, mRNA |  |  |
| Q7T3L3 | endoplasmin-like (LOC100700552), mRNA |  |  |
| A0JMI9 | ribosome-releasing factor 2, mitochondrial-like (LOC100700485), mRNA |  |  |
| Q6PGY5 | dnaJ homolog subfamily C member 21-like (LOC100690046), mRNA |  |  |
| F1QEB7 | WD repeat domain 11 (wdr11), transcript variant X2, mRNA |  |  |
| F1R4X6 | dehydrodolichyl diphosphate synthase-like (LOC100705873), transcript variant X2, mRNA |  |  |
| F1R4V5 | 28S ribosomal protein S29, mitochondrial-like (LOC100706839), transcript variant X3, mRNA |  |  |
| A0A0R4IE20 | activator of 90 kDa heat shock protein ATPase homolog 1-like (LOC100693443), transcript variant 2, mRNA |  |  |
| F6NHI0 | glucose-6-phosphate 1-dehydrogenase-like (LOC100703946), misc_RNA |  |  |
| Q6NXA1 | small glutamine-rich tetratricopeptide repeat-containing protein alpha-like (LOC100708921), transcript variant X4, mRNA |  |  |
| A0A0R4IP97 | autophagy-related protein 16-1-like (LOC100692493), transcript variant X2, mRNA |  |  |
| A0A0R4IFK5 | 28S ribosomal protein S30, mitochondrial-like (LOC100691299), mRNA |  |  |
| Q6DRD2 | proteasome (prosome, macropain) 26S subunit, ATPase, 6 (psmc6), mRNA |  |  |
| E7F2Y6 | ATP-binding cassette sub-family A member 1-like (LOC100699821), transcript variant X2, mRNA |  |  |
| E9QET5 | small cell adhesion glycoprotein-like (LOC100707778), transcript variant X1, mRNA |  |  |
| Q6DRI4 | DNA-directed RNA polymerases I and III subunit RPAC2-like (LOC100701860), transcript variant X2, mRNA |  |  |
| Q6DRH9 | transcription factor IIIA-like (LOC100709542), mRNA |  |  |
| B8A5G9 | tRNA (guanine(37)-N1)-methyltransferase-like (LOC100694441), mRNA |  |  |
| Q6NZS4 | tRNA-splicing ligase RtcB homolog (LOC100706393), transcript variant X2, mRNA |  |  |
| F1QPH6 | DEAD (Asp-Glu-Ala-Asp) box polypeptide 27 (ddx27), transcript variant X2, mRNA |  |  |
| Q1LXS2 | proteasome assembly chaperone 2-like (LOC100711738), mRNA |  |  |
| E9QEX7 | l(3)mbt-like 3 (Drosophila) (l3mbtl3), transcript variant X1, mRNA |  |  |
| B0S6A9 | WD repeat domain 3 (wdr3), transcript variant X2, mRNA |  |  |
| Q7SYL5 | PIN2/TERF1-interacting telomerase inhibitor 1-like (LOC100693022), transcript variant X1, mRNA |  |  |
| F1QK14 | TRMT1-like protein-like (LOC100711758), transcript variant X2, mRNA |  |  |
| Q4V9P9 | nucleolar protein 11-like (LOC100696496), mRNA |  |  |
| F1QUZ5 | suppressor of SWI4 1 homolog (LOC100694758), mRNA |  |  |
| E7F5Q4 | apoptosis-enhancing nuclease-like (LOC100699779), transcript variant X2, mRNA |  |  |
| F1QG82 | importin subunit alpha-1-like (LOC100698533), transcript variant X2, mRNA |  |  |
| Q5SPD1 | uncharacterized LOC100709198 (LOC100709198), transcript variant X2, mRNA |  |  |
| Q4VBK0 | ATP synthase subunit beta, mitochondrial-like (LOC100705231), mRNA |  |  |
| Q6DH30 | ectonucleoside triphosphate diphosphohydrolase 4 (entpd4), transcript variant X1, mRNA |  |  |
| F1R3W0 | TATA box-binding protein-associated factor RNA polymerase I subunit B-like (LOC100692171), mRNA |  |  |
| Q503Y4 | ovarian cancer-associated gene 2 protein homolog (LOC100700238), mRNA |  |  |
| F1QCT6 | phospholipase A2-activating protein (plaa), transcript variant X2, mRNA |  |  |
| E7F1G8 | probable ATP-dependent RNA helicase ddx6-like (LOC100698984), transcript variant X1, mRNA |  |  |
| E9QBH8 | ubiquitin-associated domain-containing protein 2-like (LOC100701243), mRNA |  |  |
| A2CEH0 | protein VPRBP-like (LOC100706874), transcript variant X3, mRNA |  |  |
| A0A0R4IBK2 | peptidyl-prolyl cis-trans isomerase E-like (LOC100711111), transcript variant X1, mRNA |  |  |
| F1QX16 | 1-acyl-sn-glycerol-3-phosphate acyltransferase delta-like (LOC100710123), transcript variant X1, mRNA |  |  |
| Q6NZU0 | heat shock 70 kDa protein 4-like (LOC100703840), transcript variant X1, mRNA |  |  |
| B2GS36 | U4/U6 small nuclear ribonucleoprotein Prp31-like (LOC100710786), mRNA |  |  |
| F1QGJ3 | perilipin-3-like (LOC100696817), transcript variant X1, mRNA |  |  |
| Q4VBR8 | proteasome subunit alpha type-2-like (LOC100694136), mRNA |  |  |
