# Supplementary material for: Transcriptome Analysis Reveals Common and Differential Response to Low Temperature Exposure Between Tolerant and Sensitive Blue Tilapia (Oreochromis aureus)
Source: Front Genet. 2019 Feb 26;10:100. doi: 10.3389/fgene.2019.00100 (PMC6399464; doi:10.3389/fgene.2019.00100)
Supplement: Supplementary file 5 [file Table_5.docx]

**Table S5.** Genes related to the glycolysis/gluconeogenesis (in the gills) and biosynthesis of amino-acids (in the liver) that were down-regulated in the tolerant fish (marked in blue) and up-regulated in the sensitive fish (marked in red), in response to low temperature challenge. P value >0.05 indicate no basal expression differences between sensitive and tolerant fish at the baseline temperature basal expression (transcripts levels at 24°C)

| **Gene** | **Uniport** | **Tissue** | **P value** |
| --- | --- | --- | --- |
| alcohol dehydrogenase class-3-like | Q6NXA6 | Gills | 0.99 |
| phosphofructokinase (pfk), transcript variant X1 | A4QNV1 | Gills | 0.72 |
| acyl-CoA synthetase short-chain family member 2 (acss2), transcript variant X1 | F1QQH3 | Gills | 0.99 |
| 6-phosphofructokinase, muscle type-like | Q66HV8 | Gills | 0.68 |
| glucose-6-phosphate isomerase-like, transcript variant 2 | Q0IJ39 | Gills | 0.99 |
| fructose-1,6-bisphosphate aldolase (ald) | Q803Q7 | Gills | 0.99 |
| pyruvate kinase (PK), transcript variant 1 | Q6DG54 | Gills | 0.99 |
| phosphoglycerate mutase 2-like | Q7T3G4 | Gills | 0.99 |
| acyl-CoA synthetase short-chain family member 1 (acss1) | F1QYS7 | Gills | 0.99 |
| glyceraldehyde-3-phosphate dehydrogenase-like | Q5XJ10 | Gills | 0.72 |
| Glyceraldehyde-3-phosphate dehydrogenase | Q5MJ86 | Gills | 0.99 |
| aldehyde dehydrogenase family 3 member B1-like, transcript variant X1 | Q90ZZ7 | Gills | 0.99 |
| glucose-6-phosphate isomerase-like, transcript variant X2 | Q7ZU30 | Gills | 0.99 |
| lactate dehydrogenase, transcript variant 1 | Q9PVK5 | Gills | 0.96 |
| 6-phosphofructokinase type C-like, transcript variant X1 | A0A0R4ITA8 | Gills | 0.99 |
| 6-phosphofructokinase type C-like, transcript variant X2 | E7FDP2 | Gills | 0.99 |
| dihydrolipoamide S-acetyltransferase (dlat), transcript variant X1 | B3DIV6 | Gills | 0.99 |
| fructose-bisphosphate aldolase C-B-like, transcript variant 1 | B2GP30 | Gills | 0.99 |
| hexokinase-2-like, transcript variant X1 | B8JKC9 | Gills | 0.99 |
| aldo-keto reductase family 1 member A1, transcript variant X1 | Q6AZW2 | Gills | 1 |
| 6-phosphofructokinase type C-like, transcript variant X2 | X1WDM7 | Gills | 0.99 |
| 6-phosphofructokinase type C-like, transcript variant X3 | E9QFJ1 | Gills | 0.99 |
| 6-phosphofructokinase type C-like, transcript variant X4 | A0A0R4ITA8 | Gills | 0.99 |
| 6-phosphofructokinase type C-like, transcript variant X6 | E7FDP2 | Gills | 0.99 |
| alpha-enolase-like, transcript variant X1 | Q6PC12 | Gills | 0.99 |
| pyruvate kinase, muscle (pkm), transcript variant X1 | A0A0R4IGP6 | Gills | 0.99 |
| fructose-1,6-bisphosphatase isozyme 2-like | A5WVL5 | Gills | 0.36 |
| triose phosphate isomerase (tpi) | Q90XG0 | Gills | 0.34 |
| hexokinase-2-like | B8JKC9 | Gills | 0.99 |
| phosphoglucomutase | F1QF00 | Gills | 1 |
| alpha-enolase-like, transcript variant X1 | Q6PC12 | Gills | 0.99 |
| phosphoglycerate mutase (pgm) | Q7SZR4 | Gills | 0.99 |
| glucose-6-phosphate isomerase-like | Q7ZU30 | Gills | 0.99 |
| phosphoenolpyruvate carboxykinase 2 (mitochondrial) (pck2) | F1R9Y5 | Gills | 0.99 |
| alpha-aminoadipic semialdehyde dehydrogenase-like, transcript variant X2 | F1QR17 | Liver | 0.97 |
| arginase-1-like | E7F8R4 | Liver | 0.99 |
| beta-enolase-like, transcript variant 1 | Q6TH14 | Liver | 0.99 |
| alanine aminotransferase 1-like | A5PLJ5 | Liver | 0.99 |
| glyceraldehyde-3-phosphate dehydrogenase-like | Q5XJ10 | Liver | 1 |
| S-adenosylmethionine synthase isoform type-1-like, transcript variant X2 | Q7ZW04 | Liver | 0.99 |
| tryptophan hydroxylase (tph), transcript variant X1 | Q7SYH6 | Liver | 0.99 |
| phosphoglycerate mutase 1-like | B8A4H6 | Liver | 0.99 |
| pyruvate kinase, liver and RBC (pklr), transcript variant X1, mRNA | Q7SXK3 | Liver | 0.99 |
| serine hydroxymethyltransferase, mitochondrial-like | A9LDD9 | Liver | 0.99 |
| alanine aminotransferase 2-like, transcript variant X2 | A0A0R4IKG8 | Liver | 0.99 |
| transketolase-like, transcript variant X1 | E7F8S4 | Liver | 0.99 |
| delta-1-pyrroline-5-carboxylate synthase-like, transcript variant X2 | A4IGC8 | Liver | 0.99 |
| fructose-bisphosphate aldolase C-B-like, transcript variant 1 | B2GP30 | Liver | 0.99 |
| arginase-2, mitochondrial-like | Q6PH54 | Liver | 0.99 |
| alpha-enolase-like, transcript variant X1 | Q6PC12 | Liver | 0.99 |
| aspartate aminotransferase, cytoplasmic-like | Q7ZUW8 | Liver | 0.99 |
| isocitrate dehydrogenase [NADP], mitochondrial-like | Q7ZUP6 | Liver | 0.99 |
| isocitrate dehydrogenase [NAD] subunit alpha, mitochondrial-like | Q7ZUJ7 | Liver | 0.99 |
| S-adenosylmethionine synthase isoform type-2-like | Q1RLT0 | Liver | 0.99 |
| phosphofructokinase, liver (pfkl) | E7F9M6 | Liver | 0.99 |
| phosphoglycerate mutase (pgm) | Q7SZR4 | Liver | 0.99 |
| phosphoserine aminotransferase-like, transcript variant 1 | Q803I7 | Liver | 0.99 |
| phosphoserine phosphatase-like | Q498V7 | Liver | 0.99 |
| pyrroline-5-carboxylate reductase 3-like, transcript variant X2 | Q5SPD7 | Liver | 0.99 |
| pyruvate carboxylase (PC), transcript variant X1 | B3DGZ9 | Liver | 0.80 |
| pyruvate kinase, muscle (pkm), transcript variant X1 | A0A0R4IGP6 | Liver | 0.99 |
| triose phosphate isomerase (tpi) | Q90XG0 | Liver | 0.99 |
