# Supplementary figures and images for: Transcriptome Analysis Reveals Common and Differential Response to Low Temperature Exposure Between Tolerant and Sensitive Blue Tilapia (Oreochromis aureus)
Source: Front Genet. 2019 Feb 26;10:100. doi: 10.3389/fgene.2019.00100 (PMC6399464; doi:10.3389/fgene.2019.00100)

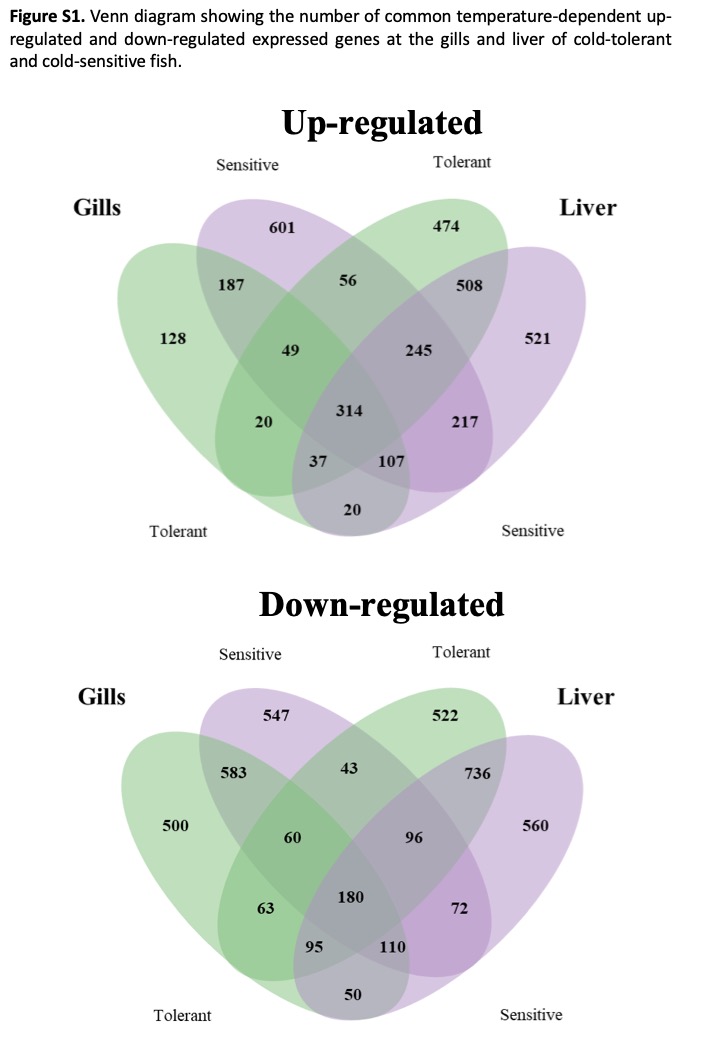

Supplement: Supplementary file 6 [file Image_1.jpg]
